# Dual-gRNA approach with limited off-target effect corrects *C9ORF72* repeat expansion in vivo

**DOI:** 10.1038/s41598-022-07746-8

**Published:** 2022-04-05

**Authors:** Xuejiao Piao, Dawei Meng, Xue Zhang, Qiang Song, Hailong Lv, Yichang Jia

**Affiliations:** 1 School of Medicine, Medical Science Building, Room D204, Tsinghua University, Beijing, 100084 China; 2 School of Life Sciences, Tsinghua University, Beijing, China; 3 Peking-Tsinghua Joint Center for Life Sciences, Beijing, China; 4 IDG/McGovern Institute for Brain Research at Tsinghua, Beijing, China

**Keywords:** Neuroscience, Diseases of the nervous system, Amyotrophic lateral sclerosis

## Abstract

*C9ORF72* GGGGCC repeat expansion is the most common genetic cause for amyotrophic lateral sclerosis and frontotemporal dementia, which generates abnormal DNA and RNA structures and produces toxic proteins. Recently, efficacy of CRISPR/Cas9-mediated editing has been proven in treatment of disease. However, DNA low complexity surrounding *C9ORF72* expansion increases the off-target risks. Here we provide a dual-gRNA design outside of the low complexity region which enables us to remove the repeat DNA in a ‘cutting-deletion-fusion’ manner with a high fusion efficiency (50%). Our dual-gRNA design limits off-target effect and does not significantly affect C9ORF72 expression. In neurons carrying patient *C9ORF72* expansion, our approach removes the repeat DNA and corrects the RNA foci in vitro and in vivo. Therefore, we conclude that our proof-of-concept design correct *C9ORF72* repeat expansion, which may have potential therapeutic value for the patients.

## Introduction

Identification of the expanded GGGGCC repeats in *C9ORF72* in both ALS (Amyotrophic lateral sclerosis) and FTD (frontotemporal dementia) provides genetic basis for the pathogenic and pathological overlaps between these two distinct neurodegenerative disorders^1,2^. The hexanucleotide repeats have explained a large proportion of ALS and FTD cases, including both familiar and sporadic ALS and FTD, underscoring the critical roles of the expanded repeats in the pathogenesis^3^.

At DNA level, the repeat expansion forms abnormal nucleotide structures, such as G-quadruplexes, which increase local epigenetic perturbations and cause haploinsufficiency in C9ORF72 expression^4–7^. Indeed, low level of C9ORF72 protein leads to immune dysregulation, which has been associated with pathogenesis of many neurodegenerative diseases^8–12^. At RNA level, the expanded GGGGCC repeats are bidirectionally transcribed into repeat RNAs, which form sense and antisense RNA foci to sequester RNA binding proteins (RBPs) and disturb normal functions of these RBPs^2,13–20^. At protein level, dipeptide repeat (DPR) proteins generated from repeat-associated non-ATG (RAN) translation contribute to the disease pathogenesis by impairing stress granule phase separation, nuclear pore protein trafficking, and heterochromatin structure^13,14,21–30^.

With the advances of genome editing approaches, especially the CRISPR/Cas9 technology, it becomes possible to correct the genetic mutations devastating to human health^31–33^. Using dual-gRNA-mediated deletion, the mutant exon of *dystrophin* in Duchenne muscular dystrophy (DMD) was removed and the muscle functions were partially restored^34–36^. The intronic location of *C9ORF72* repeat expansion makes it suitable for dual-gRNA-mediated target DNA removal in a ‘cutting-deletion-fusion’ manner and limits out-of-frame editing often introduced by exonic DNA manipulation by CRISPR/Cas9. However, low complexity of DNA region surrounding *C9ORF72* repeat site makes it almost impossible to select high quality gRNA without affecting the *C9ORF72* transcript integrity.

Here, we select two intronic gRNAs outside of the low complexity DNA region and our design removes the repeat site together with a non-coding exon (exon 1b) of *C9ORF72*. Using a sensitive and genome-wide off-target detector, we demonstrate that, indeed, the dual gRNAs we chose have much less off-target effect (at most 0.2%) than that of the gRNA (38.9%) in the low complexity DNA region. In addition, the dual gRNAs remove repeat DNA with a high fusion efficiency (~ 50%) in cell expressing Cas9 and the dual-gRNA, but have limited effect on C9ORF72 protein expression. In neurons carrying human *C9ORF72* repeat expansion, our dual gRNAs remove repeated DNA expansion and correct the expansion-generated RNA foci, a pathological hallmark shown in patients, in vitro and in vivo. Therefore, we provide a proof-of-concept solution to permanently remove *C9ORF72* repeat expansion with a high fusion efficiency and limited off-target effect, which may have potential therapeutic value to treat ALS and FTD patients carrying the *C9ORF72* repeat expansion.

## Materials and methods

### Cell culture

Primary cortical cultures were prepared from postnatal mouse pups. As previously described^37^, the cortex was cut into small pieces and digested at 37 °C for 15 min. To stop digestion, the complete medium (DMEM supplemented with 10% FBS) was applied. After digestion, the tissue suspension was filtered with a 40-µm cell strainer (Falcon) and centrifuged at 1000×g for 8 min. Cells were counted with cell counting equipment and seeded in 24-well plate at 37 °C with 5% CO_2_. After 4-h incubation, the culture medium was removed and replaced with maintenance medium, containing Neurobasal (Gibco), B27 (Invitrogen), and glutamine (Invitrogen). Anti-Tuj-1 (1;1000 mouse, Beyotime, China) immunostaining was used for characterization of the cultured neurons. HEK293, U251, and SH-SY5Y cells were grown in DMEM supplemented with 10% FBS at 37 °C with 5% CO_2_. For selecting the single clone cells, lentiviral infected cells were placed in 96-well plate by glass micropipette (Sutter Instrument). The genotypes of single clone cell lines were characterized by PCR and sanger sequencing.

### Lentiviral infection

To generate lentiviral particles expressing dual gRNAs, we inserted an additional H1 promoter into lentiCRISPR V2 (Addgene 52961) to drive the second gRNA expression. To track the lentiviral infected cells, we modified lentiCRISPR V2 by replacement of Cas9 with EGFP. For constitutively expressing Cas9, we employed lentiCas9-Blast (Addgene 52962). For virus packaging, HEK293FT cells were co-transfected with the lentiviral plasmids, pCMV-VSV-G (Addgene 8454), and psPAX2 (Addgene 12260) by polyethyleneimine (PEI) (Polysciences 23,966). 72 h after transfection, the cell culture medium was collected and ultracentifuged, and the pellet was resuspended overnight at 4 °C. Cells were infected with lentiviral particles (1 × 10^11^ VP/ml) for 48 h. If necessary, antibiotic selection was carried out to remove the uninfected cells.

### RNA foci detection in vitro and in vivo

For RNA foci detection, we employed locked nucleic acid (LNA) DNA probes (sense probe, TYE563-CCCCGGCCCCGGCCCC; antisense probe, TYE563-GGGGCCGGGGCCGGGG, Exiqon, Inc., Order catalog NO. #300,500), and performed the in situ hybridization under a RNase-free condition as described previously^11,37,38^.

For RNA foci detection in vitro, primary cortical cultures were prepared from P8 pups. After 12 days in vitro (DIV12) culture (2% B-27 and 1% GlutaMAX), the cells on the coverslip were fixed and permeabilized with 4% PFA and 0.3% Triton X-100 at room temperature for an hour. The cells were incubated with hybridization buffer, which contain 50% formamide (Biotopped), EDTA-2 × SSC (Sigma-Aldrich), 300 mM sodium chloride (pH 7.0), and 10% dextran sulfate (Biotopped), at 66 °C for an hour. After pre-hybridization, the cells were hybridized with the LNA probe (40 nM) at 66 °C for 5 h. After hybridization, the coverslips were washed with 2 × SCC in 0.1% Tween-20 at room temperature once and washed with 0.1 × SCC at 65 °C for three times. RNA foci quantification was performed as previously described^11,37,38^. For percentage of cell containing RNA foci, we only included the cells containing more than one RNA focus as previously described^17,37^. The reason is that the previous report has shown that iPSC-derived motor neuron from normal control often carries 1–2 RNA foci, which are likely nonspecific signals picked up by in situ probe^17^. Similarly, in mouse wildtype neurons a few RNA foci were detected^37^. Therefore, to more accurately estimate efficacy of our dual-gRNA approach, we did not include the neurons containing one focus into our calculation on percentage of cell containing RNA foci measurement.

For RNA foci detection in vivo, mouse brains were fixed in 4% PFA (pH 7.4) and then embedded in OCT (Sakura Tissue Tek). The frozen Sects. (10 µm) were processed with overnight hybridization. Brain sections were incubated with 0.25% Sudan black B solution (Sigma) at room temperature for 5–8 min to quenched the auto-fluorescence of lipofuscin. For in vivo and in vitro RNA foci measurement, the replicate number refers to the independent mice as the biological replicates^39^.

### Mouse

*C9ORF72*-BAC transgenic mice (C9-Tg, Stock No.023099 at JAX) were originally imported from The Jackson Laboratory and crossed to Rosa26-Cas9 knockin mice (JAX, Stock No.024858) which are constitutively expressing Cas9 endonuclease. The animal facility at Tsinghua university has been fully accredited by the Association for the Assessment and Accreditation of Laboratory Animal Care International (AAALAC) since 2014. All animal protocols were approved by the Institutional Animal Care and Use Committee (IACUC) at Tsinghua university based on Guide for the Care and use of Laboratory Animals (Eighth Edition, NHR).

### Dual-gRNA delivery in vivo

To generate recombinant adeno-associated (rAAV 1/2) particles expressing dual gRNAs, we inserted U6 and H1 promoters into pAAV-CaMKII-EGFP (Addgene 50469) to generate pAAV-gRNA1-2-CamKII-EGFP to drive the dual-gRNA expression. For rAAV packaging, the AAV plasmids were co-transfected with helper plasmids into HEK293T cells by PEI. The cells were harvested and lysed with lysis buffer, which contains 150 mM sodium chloride, 20 mM Tris–Hcl (pH = 8), 5% sodium deoxycholate (sigma #5670), and 50 U/ml Benzonase endonuclease (sigma #E1014). The viral particles were purified with HiTrap Heparin column (Sigma #5–4836) and the titer was determined by real-time quantitative PCR. For dual-gRNA delivery in vivo, mice were anesthetized with isoflurane inhalation or pentobarbital sodium injection. rAAV (> 5.0 × 10^12^ genome copies/ml) was stereotaxically injected into mouse hippocampal CA1 region at 1-month of age. The Bregma was set as 0 point and the screw of the orienteer was adjusted to y = 2.1 mm, x =  ± 1.4 mm, z-DG = 2.2 mm, z-CA1 = 1.8 mm. The virus was injected at the injection rate of 0.08 μl / min, and the injection volume was 0.6 μl per CA1 region. Two months after injection, the mice were euthanized with carbon dioxide, and the brains were harvested for RNA foci measurement. For mouse manipulation, all methods we performed in accordance with the relevant guidelines and regulations.

### Immunostaining and immunoblot

For immunostaining, primary cortical cultures were fixed with 4% PFA at room temperature for 15 min. The cells were blocked with 0.3% BSA/Triton X-100 at room temperature for 60 min, and then were incubated with the primary antibody (anti-Tuj-1, Beyotime) at 4 °C overnight. Alexa Fluor conjugated secondary antibody (ThermoFisher) was applied at room temperature for 60 min. The florescent images were captured by Leica confocal microscopy. For immunoblot, cells were lysed in RIPA buffer (50 mM Tris–HCl, pH 8.0, 150 mM NaCl, 0.25% sodium deoxycholate, 0.1% SDS, 1% NP-40, supplemented with complete protease inhibitor mixture). Blots were incubated with anti-C9ORF72 antibody (ProteinTech 66140-1) overnight at 4 °C and then HRP-conjugated secondary antibody. The band intensities were calculated by Fiji ImageJ.

### Southern blot

Genomic DNA (50 μg) from HEK293 cells infected with dual-gRNA lentivirus was digested overnight with the restriction endonucleases, and DNA fragments were separated by agarose gel. The DNA was then transferred onto Hybond-N + membrane (Amersham) by capillary equipment with 20 × SSC (30 mM sodium citrate and 300 mM sodium chloride, pH 7.0). Hybridization was performed in Church-Gilbert buffer (7% [w/v] SDS, 10 mM EDTA and 0.5 M phosphate buffer, pH 7.2) at 65 °C overnight with [α-^32^P]-labeled (Perkin-Elmer) DNA probe (Rediprime II System, Amersham). The membrane was washed with 2 × SSC and 0.1% SDS twice at 65 °C for 20 min each, and then washed with 1 × SSC and 0.1% SDS for 20 min. Signals were developed on X-ray film for 3 days at −70 °C. The band intensities were calculated by Fiji ImageJ.

### GUIDE-seq and data analysis

For GUIDE-seq, 500 ng dual gRNAs (gRNA1-2 or gRNA3-b) and 50 pmol double-stranded oligodeoxynucleotides (dsODN) were transfected into HEK293 cells constitutively expressing Cas9. Cells transfected with dsODN alone were employed as a negative control. 48 h after transfection, cells were collected and genomic DNA was isolated using TIANamp Genomic DNA Kit (TIANGEN Biotech). The genomic DNA was sheared by CovarisS2 to yield ~ 500 bp fragments. The DNA fragments were purified by AMPure beads (Beckman), and the GUIDE-seq library was constructed as previously described^40^. To adapt our sequencing platform, we did some modification on the Y-adapter (Supplementary Fig. 3). The resulting samples were sequenced on HiseqXten-PE150 (Illumina) and paired-end reads with 150-nt lengths were obtained (Supplementary Fig. 3). For data analysis, the raw data went through three major steps, including preprocessing, alignment, and filtering, to achieve the reliable dsODN integration sites (Supplementary Fig. 4). In preprocessing step, adapter reads were removed and low quality nucleotides were trimmed. Paired-end reads containing UMI in Read1 and dsODN tag in Read2 were collected for further processing while unpaired reads were removed. Paired-end reads containing the same UMI and genome sequence were considered as PCR duplicates and removed. In alignment step, the UMI and dsODN tags were cropped to generate clean reads, which were aligned to the human genome by BWA aligner^41^. The improperly aligned read pairs were removed. Bedtools was used to count the aligned reads at each locus^42^. In filtering step, we allowed 5 mismatches and generated candidate off-target loci for further assessment based on Cas-OFFinder annotation. Those potential off-target sites found in negative control were not considered as real off-target sites but DNA break hot spots. Finally, the gRNA on-target and off-target sites that were supported by unique reads at different chromosomes or from at least two out of our three independent experiments were considered to be reliable. The raw data was public available via the link: https://pan.baidu.com/s/1k0hUrcAGFz7xCAELtAnwuw (with the password: tny3).

### Targeted deep sequencing of potential off-target sites

Genomic sequence containing the potential off-target sites identified by GUIDE-seq were PCR-amplified by 20 cycles with 2 × KAPA HiFi HotStart ReadyMix (Kapa Biosystems). Adapters containing the P5 and P7 sequences were incorporated into the PCR products by another 15 PCR cycles. The final Illumina libraries were purified with Ampure XP beads (Beckman) and quantified by Qubit 3.0 Flurometer (Life Technologies). Agilent 2100 Bioanalyzer (Agilent Technologies) was used to characterize the size and quality of the libraries. After Illumina sequencing, clean reads were extracted and aligned to the human genome hg19 using BWA. Samtools was used to count the variant reads number at each targeted sites.

### RNA-Seq

HEK293 cells expressing Cas9 were infected with dual-gRNA1-2 lentivirus. After puromycin selection, total RNA was extracted and dissolved in nuclease-free water, treated with DNase (Ambion, Thermo Fisher Scientific), and quantified by Qubit RNA Assay Kit (Thermo Fisher Scientific). Agilent 2100 Bioanalyzer (Agilent Technologies) was employed to check the quality of RNA. Total RNA (3 μg) was applied for poly(A) mRNA purification by using oligo-d(T) magnetic beads (S1419S, NEB). RNA fragmentation, cDNA synthesis, terminal repair, A-tailing, and adapter ligation were performed by using RNA library prep kit (E7530L, NEB) as manual instructions. DNA products were cleaned using AMPure XP beads (Beckman). Library quality was checked by Agilent 2100 Bioanalyzer and quantified by real-time PCR. Sequencing was performed on the Illumina HiSeq platform. The clean reads were aligned to human genome hg38 using HISAT2, and the mRNA expression level was quantified by an R package DESeq2. The C9ORF72 splice junctions were visualized by Integrative Genomics Viewer (IGV).

### Statistical analysis

The sample size was computed based on the experimental models and previous studies when the study was designed. We set the positive and negative controls for all our experiments and included or excluded the data points based on the performance of our positive and negative controls. We employed SPSS to assess whether the data met the assumptions of the statistical approach, and if the assumptions were not met, we adjusted to other statistical approach. Biological replication is the parallel measurement of biologically different samples to capture random biological variations, which differs from the technical duplication that is repeated measurements of the same sample, representing multiple independent measurements. In immunoblot, southern blot, and GUIDE-seq assays the replicate number refers to independent cell cultures. For in vivo and in vitro* RNA foci* measurement, the replicate number refers to the independent mice. All the values shown in this manuscript are presented as mean ± SEM and the statistical details are described in the Figures and the corresponding legends.

All methods were performed in accordance with The National Natural Science Foundation of China (NSFC) guidelines and regulations. The study is reported in accordance with ARRIVE guidelines.

## Results

### Dual-gRNA design for removal of human C9ORF72 repeat site

**Figure 1:**
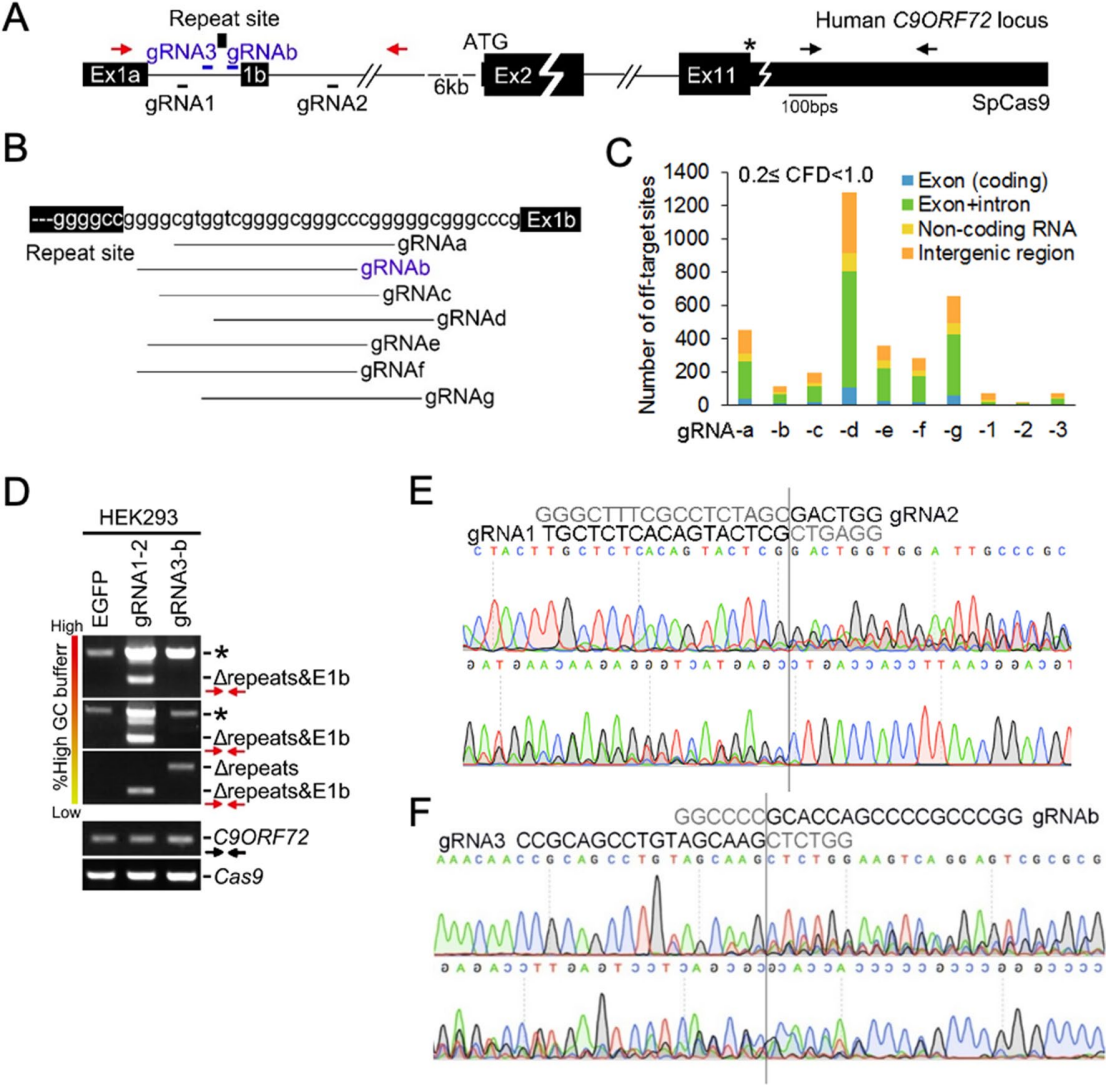
Our dual gRNAs remove the *C9ORF72* repeat site in human cell. (**A**) The *C9ORF72* genome structure (GRCh37/hg19) and our dual-gRNA design (gRNA1-2 pair or gRNA 3-b pair) for removal of the *C9ORF72* repeat site. The gRNAs described here are SpCas9-based. The primers flanking the gRNA editing sites labeled as red arrows used for genotyping the removal. The primers in last exon (exon 11, Ex11) labeled as black arrows used for detecting *C9ORF72.* (**B**) All available gRNAs lie between the repeat site and exon 1b (Ex1b). The distance between the repeat site and exon 1b is 35bps. The gRNAb is in antisense orientation, and the rest of gRNAs are in sense orientation. (**C**) Off-target summary of gRNAs we examined by using an online gRNA design tool (https://portals.broadinstitute.org/gpp/public/analysis-tools/sgrna-design). The off-target details are shown in Supplementary Table 1. (**D**) Genomic DNA PCR for detecting the removal of repeat site by gRNA1-2 and gRNA3-b. The HEK293 cells constitutively expressing Cas9 were infected with the lentiviral control, gRNA1-2, and gRNA3-b, respectively (also see Supplementary Fig. 2). The repeat site deletion (ΔRepeats) or repeat site plus exon1b (E1b) deletion (ΔRepeats + E1b) appeared in cells infected with gRNA1-2 or gRNA3-b. Due to the high GC content of the DNA region we amplified, PCR amplicons containing the repeat site and its neighboring sequences (labelled as asterisk) did not appear in control group in the PCR condition with low amount of high GC buffer. High GC buffer condition: from red to yellow, usage of high GC buffer from high to low %. The amplicons of *C9ORF72* last exon were used for PCR reaction control. The primers used here were illustrated in (**A**). (**E** and **F**) The repeat deletion bands (Δ) shown in (**D**) were applied for both forward and reverse Sanger sequencing. The editing site fusions took place upstream of the PAM (the protospacer adjacent motif, NGG) sites.

In order to remove the expanded GGGGCC repeats in the intron of *C9ORF72*, we analyzed the genomic sequences of human *C9ORF72* (Fig. 1A). Three times of GGGGCC (repeat site) are found 162-bp downstream of exon 1a and 35-bp upstream of exon 1b, consistent with the previous repeat site description (Fig. 1A)^1,2^. By using two online gRNA designers^43,44^, we identified two gRNAs, gRNA1 and gRNA3, as upstream gRNAs with low predicted off-target effect in the 162-bp region between the exon 1a and the repeat site (Fig. 1A and Supplementary Table 1). For downstream gRNA, the ideal location is in the 35-bp region between repeat site and exon 1b, which pairs with the upstream gRNA to remove the repeat expansion without disturbing exon 1b. However, the 35-bp region is a low complexity region and all the gRNAs we identified in this 35-bp region have high predicted off-target effect. Among them, gRNAb is scored the best (Fig. 1B, C, Supplementary Table 1). In order to avoid the potential off-target effect of the downstream gRNAs, we sought DNA region downstream of exon 1b and identified gRNA2, which is close to exon 1b and has low predicted off-target effect (Fig. 1A, C, Supplementary Table 1). Therefore, we ended up with two designs: (1) gRNA1 and gRNA2 pair (gRNA1-2), which removes the repeat site together with the exon 1b with low predicted off-target effect; (2) gRNA3 and gRNAb pair (gRNA3-b), which removes the repeat site and keeps the exon 1b intact with high predicted off-target effect (Fig. 1A).

### The dual gRNAs remove C9ORF72 repeat site in human cell

To test our designs, we employed human HEK293 cell that contains 2 ~ 3 GGGGCC repeats (Supplementary Fig. 1A) and ensured that our gRNA target sequences are present in the genomic DNA (Supplementary Fig. 1B). Besides HEK293 cell, the target sequences of gRNA1, 2, and b are present in the other 11 human cell lines we examined (Supplementary Fig. 1C). We noticed a SNP (single nucleotide polymorphism) in gRNA3 target sequences in 3 out of 12 human cell lines we examined (Supplementary Fig. 1C and 1D), suggesting a SNP hot spot in the target sequences, which may need additional design for future precise editing.

To express two gRNAs in the same cell with high efficiency, we employed previously reported dual-gRNA expression and lentiviral delivery systems^45,46^ (Supplementary Fig. 2). The HEK293 cells constitutively expressing Cas9 were infected with the lentiviral control, gRNA1-2, and gRNA3-b, respectively. To examine deletion of the repeat site, which and surrounding sequences of which are GC-rich, we used primers flanking the repeat site and performed genomic DNA PCR with high GC PCR buffer (Fig. 1A, D). With the buffer, we amplified the repeat site and its surrounding sequences in the HEK293 cells infected with the lentiviral control, gRNA1-2, and gRNA3-b. In addition, a deletion of repeat plus exon1b (E1b) (ΔRepeats + E1b) band appeared in HEK293 cells infected with gRNA1-2 with the similar PCR conditions. Due to a small deletion (ΔRepeats) by gRNA3-b, we were not able to distinguish ΔRepeats with the PCR amplicons with repeat site. However, when we lowered amount of high GC buffer in the PCR reactions, we were able to see the clear ΔRepeats or ΔRepeats + E1b bands in the HEK293 cells infected with gRNA1-2 or gRNA3-b but not in that of control virus, suggesting that a ‘cutting-deletion-fusion’ of repeat site takes place in the cells by gRNA1-2 and gRNA3-b. Indeed, the editing site fusions were evidenced at the Cas9 cutting sites by Sanger sequencing (Fig. 1E, F). Therefore, we conclude that our approach is able to remove *C9ORF72* repeat site in human cell.

### Limited off-target effect of gRNA1-2 measured by GUIDE-seq

**Figure 2:**
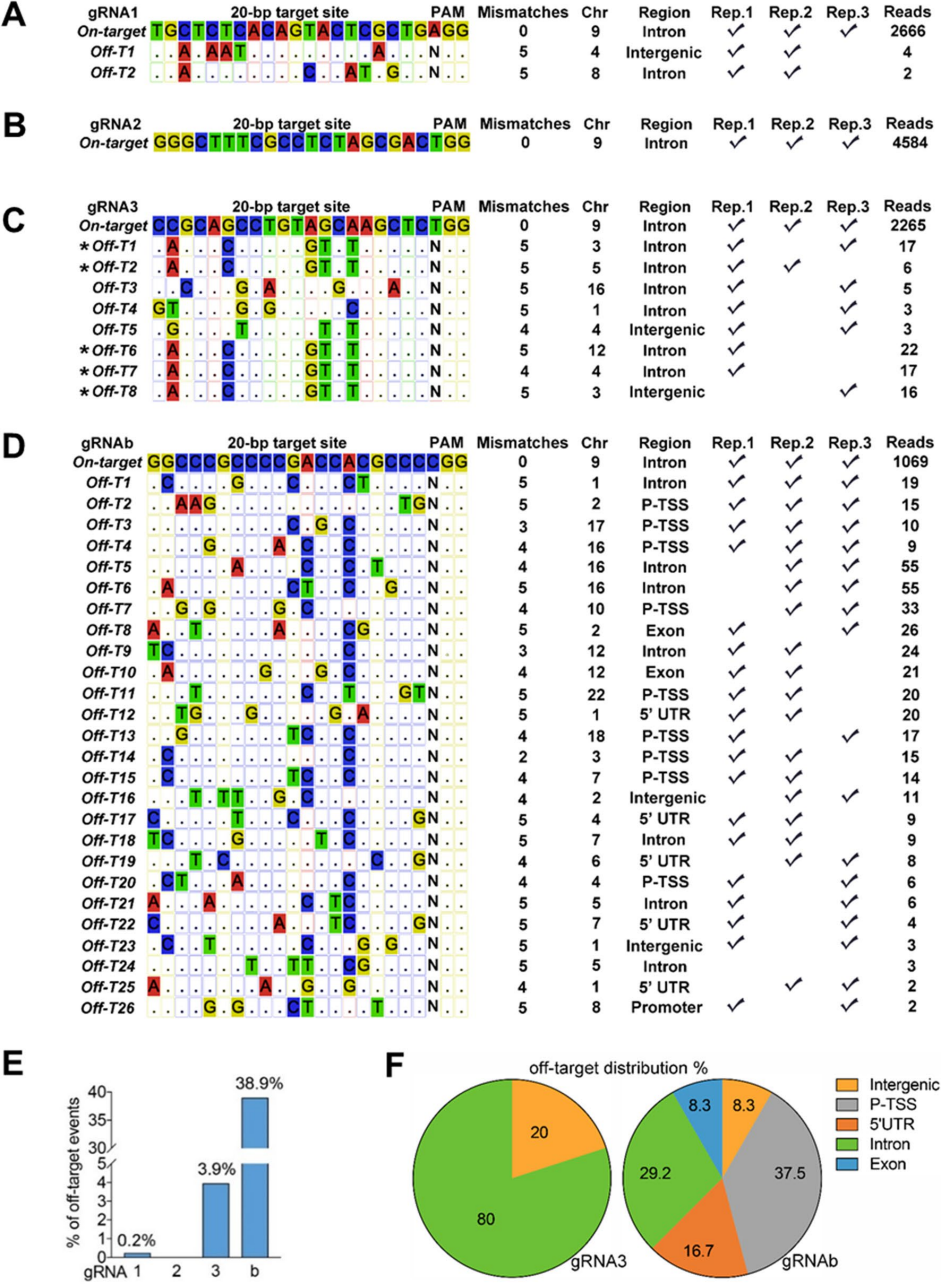
Limited off-target effect of gRNA1-2 measured by GUIDE-seq. (**A**-**D**) Off-target sites identified by GUIDE-seq. Mismatches are highlighted in colors. In c, asterisks indicate that the same off target sequences in different chromosomes. To increase reliability of off-target events detected by GUIDE-seq, we included the events that were supported by unique reads at different chromosomes or from at least two out of our three independent experiments (Supplementary Fig. 3). (**E**) Off-target percentage was estimated by ratio of off-target reads to on-target reads. (**F**) Off-target site distribution of gRNA3 and gRNAb. In (**A**-**D)**, mismatches in protospacer are no more than 5. P-TSS, region between promoter and TSS (transcription start site). 5’UTR, 5’ untranslated region.

Given that off-target effect introduced by CRISPR/Cas9 is a major risk factor for its application in human disease therapy, limited off-target effect of our gRNAs is essential for their future applications. To identify potential off-target sites of our gRNAs, we employed both computational prediction and experimental identification approaches. In agreement with other online off-target predictors (Supplementary Table 1), Cas-OFFinder, a fast and versatile algorithm^47^, predicted much more off-targets in gRNA3-b pair than in gRNA1-2 pair when we allowed mismatches (up to 7 mismatches) in the protospacer region (Supplementary Table 2). To experimentally identify off-target genomic loci of our gRNAs, we employed GUIDE-seq, a unbiased and sensitive genome-wide off-target site identification approach^40^. We delivered our dual gRNAs and the oligodeoxynucleotide (dsODN) that incorporates into cutting site induced by CRISPR/Cas9 into HEK293 cells constitutively expressing Cas9 (Supplementary Fig. 3A). Indeed, thousands of unique reads from three individual experiments support on-target integration of dsODN of each one of our gRNAs by GUIDE-seq (Fig. 2A-D). The on-target integration was further confirmed by genomic DNA PCR (Supplementary Fig. 4).

To extract reliable off-target events detected by GUIDE-seq, we included the events that were supported by unique reads or from at least two out of our three independent experiments (Fig. 2A-D and Supplementary Fig. 3B). The off-target rate was estimated by percentage of reliable off-target reads divided by total reads. In contrast to high off-target rate of gRNA3 (3.9%) and gRNAb (38.9%) identified by GUIDE-seq (Fig. 2C-E), only two off-target events (0.2%) were detected in the cells treated with gRNA1 and no reliable off-target event was detected for gRNA2 (Fig. 2E). For gRNA3 off-target sites, a majority of them (80%) are located in the introns and the rest of them (20%) are in the intergenic region (Fig. 2F). Because the target sequences of gRNAb are GC-rich, many of gRNAb off-target sites are located in the region between promoter and transcription start site (P-TSS, 37.5%) and 5’UTR (16.7%). Some gRNAb off-target sites hit protein coding exons (8.3%), suggesting that the off-target effect by gRNAb leads to functional impairments of these genes.

To confirm the off-target events identified by GUIDE-seq, we performed targeted deep sequencing of Off-T1 and Off-T2 of gRNA1 and 8 potential (P) off-target sites (up to 3-mismatches) of gRNA1 and gRNA2, respectively (Supplementary Fig. 5 and Supplementary Table 2). HEK 293 cells constitutively expressing Cas9 were transfected with gRNA1 or gRNA2 and applied for the targeted deep sequencing. Meanwhile, the cells transfected with GFP were employed as a negative control. Although at least 2.0 × 10^5^ coverage of each sites were achieved in the cells transfected with gRNA1 or gRNA2, we did not observe differential indel rate between gRNA-transfected and GFP-transfected cells, suggesting that Off-T1 and Off-T2 of gRNA1 and potential off-target sites of gRNA1 (P1–P7) and gRNA2 (P1) are probably not caused by gRNA1- and gRNA2-mediated DNA editing. Taken together, our online prediction and experimental results support limited off-target effect of our gRNA1-2 design, but high off-target risks of gRNAb, which may limit its future clinical application.

### Deletion of repeat site together with exon 1b has limited effect on C9ORF72 protein expression

**Figure 3:**
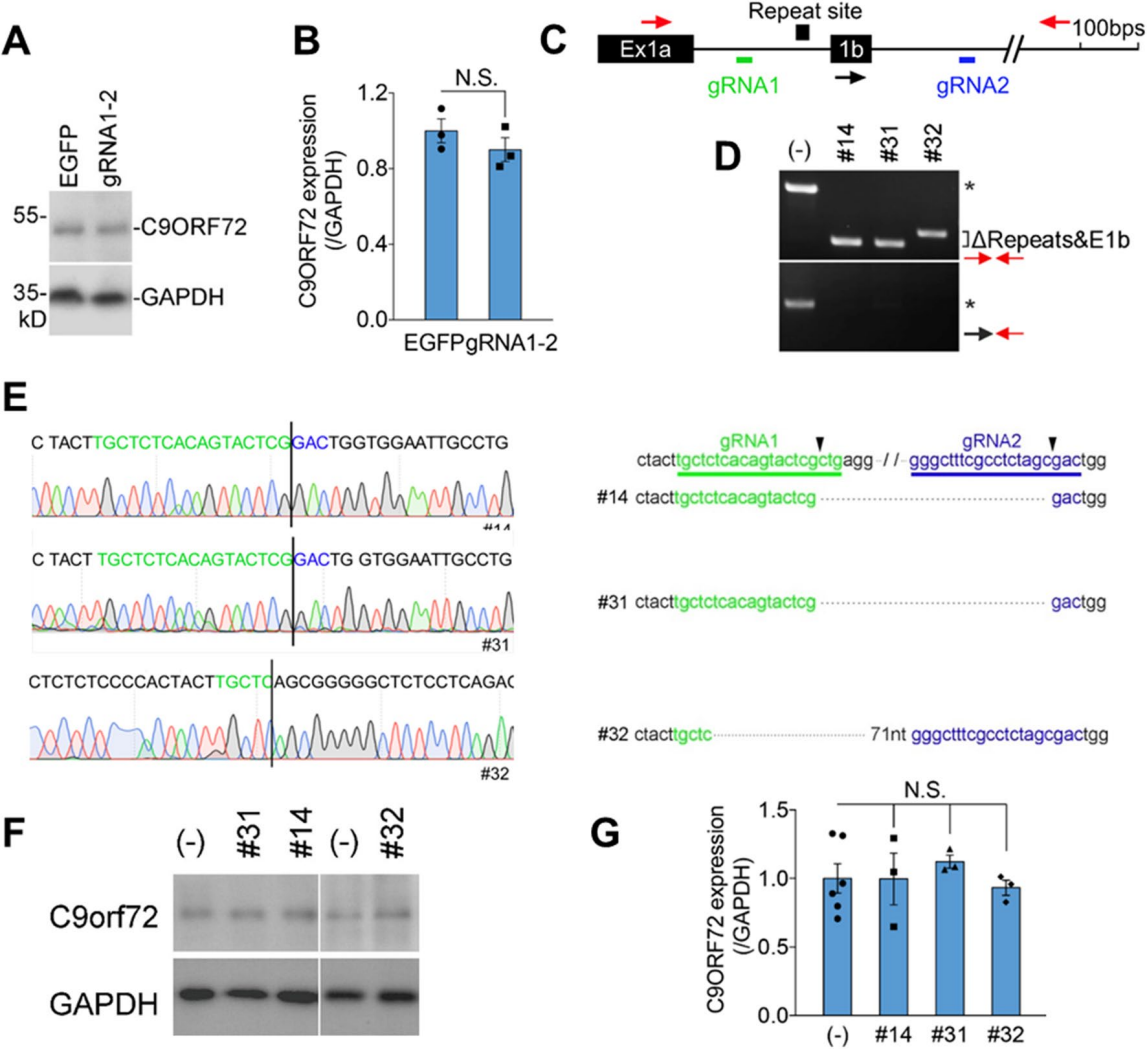
Deletion of repeat site and exon 1b by gRNA1-2 has limited effect on C9ORF72 protein expression. (**A**) Representative image of C9ORF72 immunoblot from HEK293 cells constitutively expressing Cas9 and infected with control (EGFP) or gRNA1-2 lentiviral particles. GAPDH served as loading control. (**B**) Relative expression level of C9ORF72 protein (normalized to GAPDH) shown in (**A**). Protein lysates from three independent infections. (**C**) The genome structure of *C9ORF72* exon 1a (Ex1a) and exon 1b (1b) and locations of gRNA1 and gRNA2. Primers used for screening for HEK293 single cell clones with repeat site and 1b deletion. (**D**) Identification of single cell clones with deletion of the repeat site and 1b (ΔRepeats + E1b) by gRNA1-2. Asterisk indicated the normal PCR bands that appeared in non-editing control (-). Upper panel, primers flanking the repeat site and exon 1b detected smaller bands (ΔRepeats + E1b) in three single cell clones (#14, 31, and 32), however, the normal PCR band (*) did not appear in these lines. Lower panel, PCR band with exon 1b (*) appeared in non-editing control (-) but not in these single cell clones. PCR condition in upper panel, with high GC buffer. (**E**) The smaller bands (ΔRepeats + E1b) shown in (**D**) were applied for Sanger sequencing (Left). Sequence alignment represents the exact deletions in these three single cell clones (Right). The conventional SpCas9 cleavage sites (3 neucleotides upstream of PAM) of gRNA1 and gRNA2 were labeled (black arrow head). (**F**) Representative images of C9ORF72 immunoblot from the control (-) and #14, 31, and 32 single cell clones. (**G**) Statistical analysis of C9ORF72 relative expression level shown in (**F**). The protein lysates from at least three biological replicates. In (**B**) and (**G**), the values are presented as mean ± SEM (*n* ≥ 3). N.S., no statistical significance (t-test or ANOVA, SPSS).

Haploinsufficiency of *C9ORF72* in C9-ALS/FTD patients implies that loss-of-function of *C9ORF72* may contribute to disease pathogenesis^1,2^. In fact, *C9orf72* deficient mice showed abnormal immune response^8–10^. Given that our gRNA1-2 design removes both the repeat site and exon 1b, we then asked whether the removal affects the C9ORF72 protein expression. Using a validated C9ORF72 antibody (Supplementary Fig. 6), we demonstrated that the relative C9ORF72 level was slightly decreased in HEK293 cells infected with gRNA1-2 (0.9 ± 0.06) compared to non-editing control (1.0 ± 0.06) with no statistical significance (Fig. 3A, B). In addition, our RNA-Seq data suggest no significant *C9ORF72* level change in HEK293 cells infected with gRNA1-2 lentivirus, compared to HEK293 control cells (Supplementary Fig. 7A). Both intron 1 and intron 2 are efficiently spliced in these cells infected with gRNA1-2, suggesting exon 1b removal does not severely change the *C9ORF72* expression and splicing (Supplementary Fig. 7).

To examine whether gRNA1-2 affect C9ORF72 protein expression in cells derived from human brain, we employed U251 and SH-SY5Y, the human glioblastoma and neuroblastoma lines. We infected these two cell lines with Cas9-expressing lentivirus and established the U251 and SH-SY5Y cell lines constitutively expressing Cas9. After we infected these two cell lines with gRNA1-2 lentiviral particles, the removal of repeat site and exon 1b was evidenced by genomic DNA PCR with low amount of high GC buffer (Supplementary Fig. 8A), similar to what we observed in HEK293 cells (Fig. 1D). In addition, the editing site fusions were confirmed by Sanger sequencing (Supplementary Fig. 8B and 8C). As we observed in HEK293 cells (Fig. 3A), we did not observe significant change of C9ORF72 protein expression in these two cell lines expressing gRNA1-2 compared to that of control (Supplementary Fig. 8D and 8E).

To further examine the effect of gRNA1-2 on C9ORF72 protein expression, we established three independent HEK293 cell lines derived from a single edited cell by gRNA1-2 infection. Our genomic DNA PCR suggested that both alleles of repeat site and exon 1b were removed by gRNA1-2 in these three single cell clones (Fig. 3C, D), which was further confirmed by Sanger sequencing (Fig. 3E). In comparison with the non-editing control, no significant C9ORF72 protein expression level change was evidenced in these single cell clones (Fig. 3F, G). Taken together, our results indicated that removal of repeat site and exon 1b by our dual gRNAs has limited effect on C9ORF72 protein expression at least in cultured human cell lines, including HEK293, U251, and SH-SY5Y cells.

### Our gRNA1-2 lead to high fusion efficiency at their editing sites in human cell

**Figure 4:**
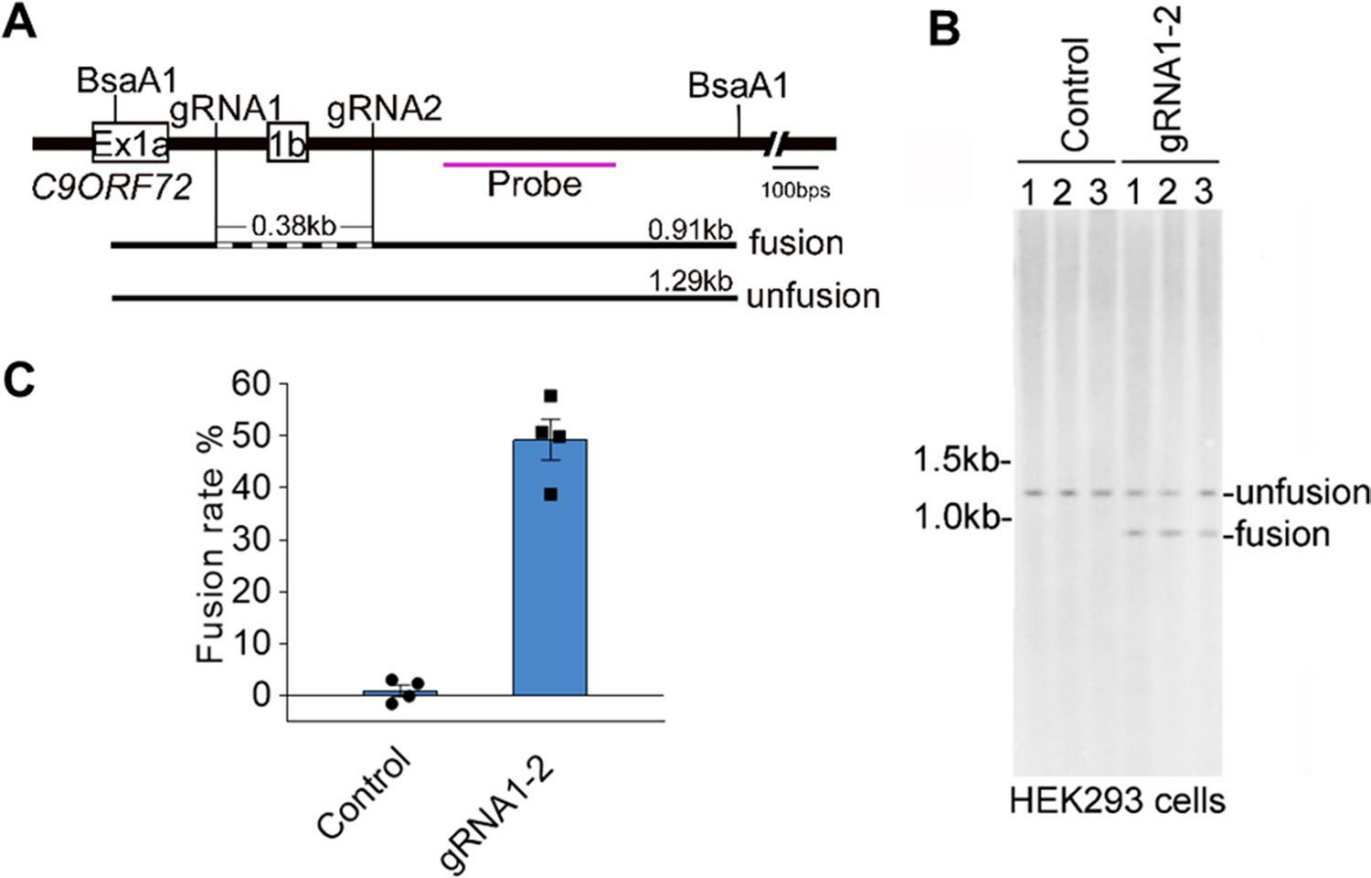
The fusion efficiency of gRNA1-2 editing sites in the HEK293 cells. (**A**) The experimental design for detecting the fusion efficiency by Southern blot. The BsaA1 enzyme was used to fragment the genomic DNA. The Southern blot probe was labeled in pink. (**B**) Genomic DNA from the HEK293 cells infected with control or gRNA1-2 lentivirus (Supplementary Fig. 2) was applied for the Southern blot. The fusion bands were present in the cells infected with gRNA1-2 but not in the controls from three biological replicates. (**C**) The fusion efficiency was estimated by the ratio of fusion to total (fusion + unfusion) band densities. Genomic DNA from 4 biological replicates. The values are presented as mean ± SEM (*n* = 4).

The removal of repeat expansion depends on the fusion of the gRNA1 and 2 editing sites. Therefore, high fusion efficiency will lead to high chance of repeat expansion removal and better therapeutic outcome. To examine the fusion efficiency, we infected HEK293 cells constitutively expressing Cas9 with gRNA1-2 lentiviral particles and performed Southern blot (Fig. 4A, B). The unfusion bands appeared in both cells infected with control or dual-gRNA lentiviral particles. However, the fusion band only appeared in the cells infected with dual gRNA1-2. These results demonstrated that the fusion results from dual-gRNA-mediated editing, which does not always lead to fusion. Next, we calculated the fusion rate by measuring the ratio of fusion to total band intensities, and revealed that the repeat site removal rate reached to 49.5 ± 5.8% in the HEK 293 cells (Fig. 4C). To further test the fusion efficiency of gRNA1-2, we isolated single clones from the infected HEK293 cells and examined the fusion by PCR. Of the total 38 clones positive for the PCR band(s), 13 clones showed two allele fusion and 13 showed single allele fusion, thus the calculated fusion rate is 51.3% (39/76) (Supplementary Fig. 9). Therefore, we concluded that our designed gRNA1-2 lead to about 50% fusion at their editing sites, at least in the HEK293 cells we examined, which constitutively expressed Cas9 and were infected with our dual-gRNA.

### Our dual gRNAs remove C9ORF72 repeat expansion and correct the repeat-produced RNA foci in primary cortical cultures

**Figure 5:**
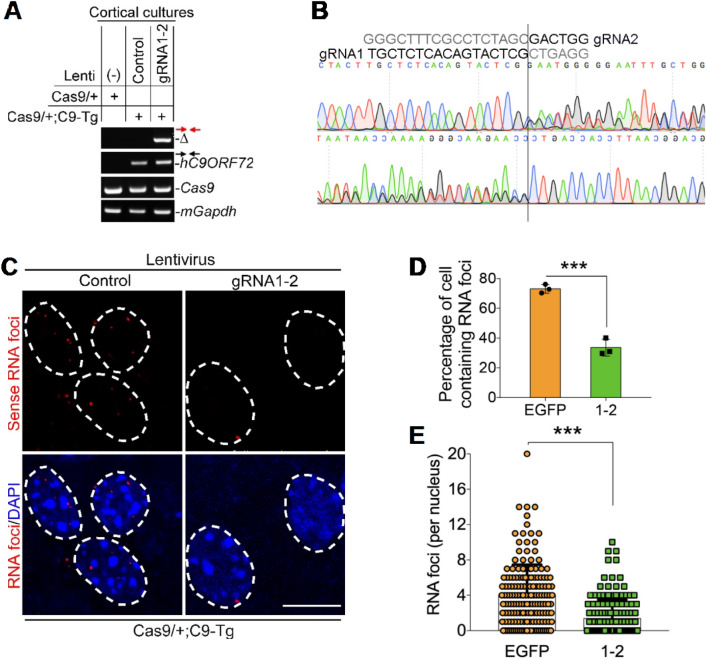
Our designed gRNA1-2 remove *C9ORF72* repeat expansion and correct *C9ORF72* sense RNA foci in primary cortical cultures. (**A**) The primary cortical neurons cultured from Cas9/ + or Cas9/ + ; C9-Tg mice were infected with control or gRNA1-2 lentiviral particles (Supplementary Fig. 2). The Cas9/ + mouse constitutively expresses *Cas9* (PMID: 25,263,330) and the *C9ORF72*-BAC transgenic mouse (C9-Tg) carries expanded GGGGCC repeats from patient (C9-Tg mouse line 112, PMID: 26,637,796). The editing site fusions (Δ) were detected by primers shown in Fig. 1A and the PCR conditions (low amount of high GC buffer) were shown in Fig. 1D. The amplicons of *C9ORF72* last exon only appeared in the neurons derived from mouse carrying C9-Tg (primers shown in Fig. 1A). Mouse *Gapdh* served as PCR reaction control. (**B**) The deletion band (Δ) shown in (**A**) was applied for both forward and reverse Sanger sequencing. The editing site fusions took place upstream of the PAM sites. (**C**-**E**) gRNA1-2 significantly reduced the sense RNA foci produced by the *C9ORF72* repeats in the primary cortical cultures derived from Cas9/ + ; C9-Tg mice. Control, control lentiviral particles. Both the percentage of cells containing foci (**D**) and the number of foci per nucleus (**E**) were significantly reduced in the dual-gRNA treatment groups. In (**C)**, the dotted line circled nuclei of cultured neurons infected with indicated lentiviral particles. In (**D** and **E)**, the RNA foci counts from three biological replicates. In (**D)**, the values are presented as mean ± SEM (*n* = 3). In (**E)**, we employed “superplot”^70^ to transparently show the spread in the number of foci per nucleus, and the values are presented as mean ± SD. ***p < 0.001 (t-test, SPSS). In (**C)**, scale bar, 10 μm.

To examine whether our dual-gRNA strategy is able to remove patient expanded GGGGCC repeats, we cultured the cortical neurons derived from *C9ORF72*-BAC (C9-Tg) transgenic mouse (line 112), which carries 100–1000 repeat expansion in size^37^. The existence of our gRNA1-2 target sequences was confirmed in the C9-Tg but not in the non-transgenic mice, indicating that the target sequences came from the BAC *C9ORF72* transgene (Supplementary Fig. 10). We then crossed the C9-Tg mouse with Rosa26-Cas9 knock-in mouse^48^ to ensure constitutive expression of Cas9 in the brain and in its derived neurons. Indeed, the editing site fusion appeared in the primary cultures infected with the lentiviral particles expressing the gRNA1-2 but not empty control (Fig. 5A). Like HEK293 cells, the fusion took place at the Cas9 cutting sites, confirming the removal of repeat expansion by our dual gRNAs (Fig. 5B).

We next investigated whether the pathology produced by the *C9ORF72* repeat expansion is able to be corrected by our dual-gRNA approach. Similar to a previous report^37^, RNA foci were detected in the primary cortical cultures derived from C9-Tg but not from wild type mice (Supplementary Fig. 11). With our high lentiviral infection rate (94.8 ± 2.7%) in our primary cultures and efficient GC repeats removal (Supplementary Fig. 12), we expected a high foci correction efficacy in these infected primary cultures. Indeed, about half of sense foci were corrected by expression of gRNA1-2, which was evidenced by the percentage of cells containing the foci and the numbers of foci in the given cells (Fig. 5C-E). Therefore, we concluded that our approach is able to remove the *C9ORF72* repeat expansion and correct the repeat-produced RNA foci, one of the molecular and pathological hallmarks shown in the patients, in the primary cortical cultures carrying patient repeat expansion.

### Our dual gRNAs remove C9ORF72 repeat expansion and correct the repeat-produced RNA foci in C9-Tg brain

**Figure 6:**
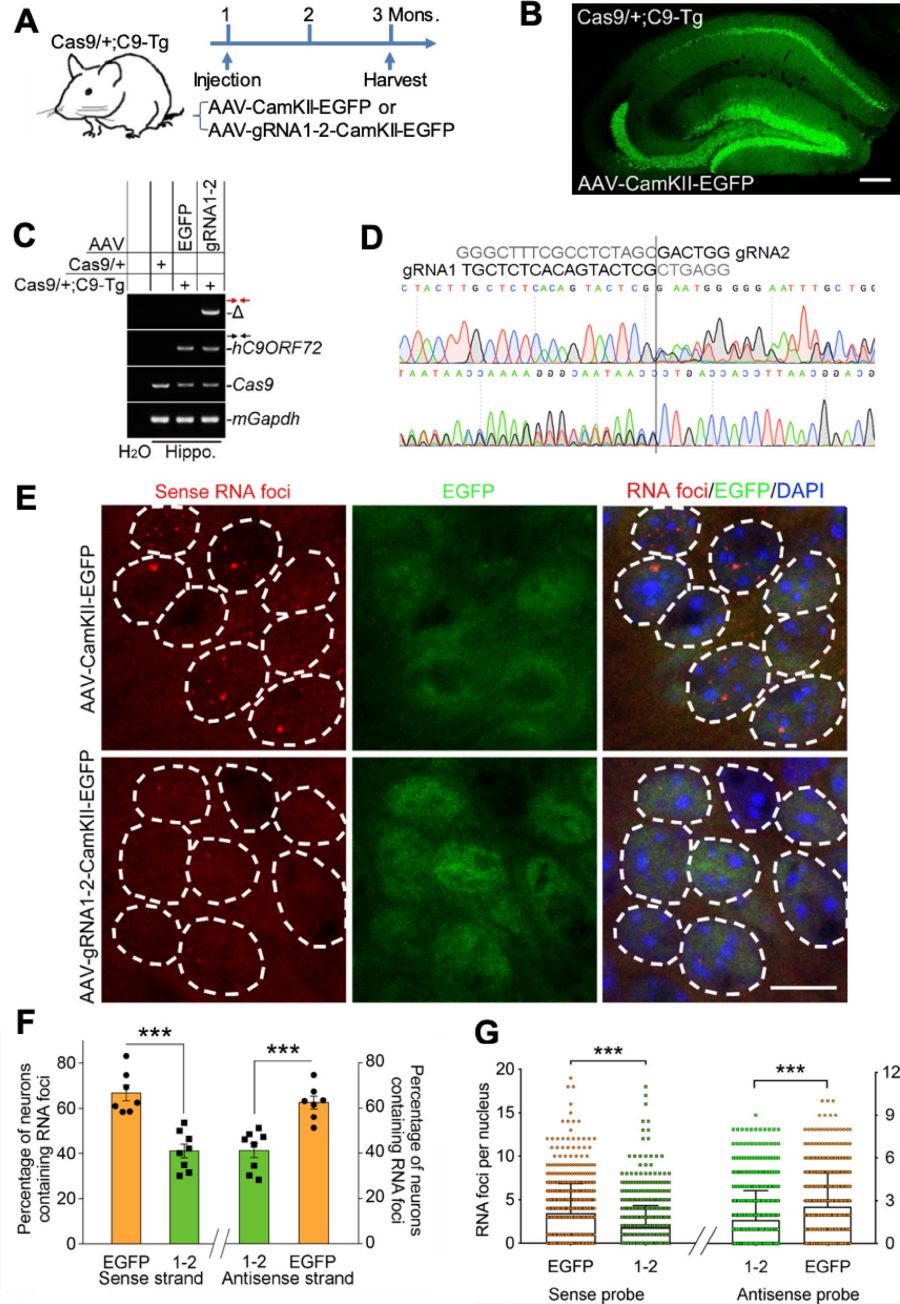
AAV-based dual gRNAs remove the *C9ORF72* repeats and correct both sense and antisense RNA foci in vivo. (**A**) The experimental procedure for removal of the *C9ORF72* repeats in vivo. The AAV-CamKII-EGFP or AAV-gRNA1-2-CamKII-EGFP (Supplementary Fig. 14) was injected into hippocampi of the Cas9/ + ; C9-Tg mice at one month of age and the infected hippocampi were harvested two months after injection. (**B**) The representative image of hippocampus infected with AAV-CamKII-EGFP. (**C** and **D**) The band (Δ) of fusion at two editing sites only appeared in the hippocampus (Hippo.) of the Cas9/ + ; C9-Tg mouse injected with AAV-gRNA1-2-CamKII-EGFP (gRNA1-2) but not in that with AAV-CamKII-EGFP (EGFP) or PCR negative control (H_2_O). The PCR conditions and primers as shown in Fig. 5A. The band (Δ) shown in (**C**) was applied for both forward and reverse Sanger sequencing (**D**). The editing site fusions were confirmed at the upstream of the PAM sites. (**E**) The AAV-CamKII-EGFP- or AAV-gRNA1-2-CamKII-EGFP-infected hippocampal CA1 regions of Cas9/ + ;C9-Tg mice were applied for the RNA foci measurement. The AAV-mediated EGFP expression was used to trace the infected region and neurons. The dotted line circled nuclei of CA1 neurons infected with indicated AAVs. (**F** and **G**) The percentage of infected neurons containing the RNA foci (**F**) and the number of foci per nucleus (**G**) were measured. For both sense and antisense RNA foci detection, animal numbers injected with AAV-CamKII-EGFP (*n* = 7) or AAV-gRNA1-2-CamKII-EGFP (*n* = 8). In **F**, the values are presented as mean ± SEM. In **G**, we employed “superplot” to show the spread in the number of foci per nucleus, and the values are presented as mean ± SD. ** *p* < 0.01, ***p < 0.001 (t-test, SPSS). Scale bar in b, 200 μm; in e,10 μm.

With limited off-target effect, limited effect on C9ORF72 protein expression, high fusion rate, and RNA foci correction in vitro, we next asked whether our dual gRNAs are able to correct repeat-produced pathology in vivo. In agreement with a previous report^37^, expression of repeat expansion generated widespread sense and antisense RNA foci in hippocampal CA1 pyramidal neurons at both 1- and 3-month old C9-Tg mice but not in that of 3-month old wildtype mice (Supplementary Fig. 13). We then crossed those C9-Tg mice with Rosa26-Cas9 knock-in mice to obtain the offsprings expressing both *C9ORF72* repeat expansion and Cas9 (Fig. 6A). In these double positive mice, we injected AAV expressing dual gRNA1-2 driven by H1/U6 promoters and EGFP driven by CamKII promoter, a neuronal promoter, which enabled us to monitor the gRNA-targeted neurons in hippocampal CA1 region (Fig. 6A and Supplementary Fig. 14). The injection of AAV expressing EGFP driven by CamKII promoter (AAV-CamKII-EGFP) alone was used as non-editing control. After two month of injection, we achieved high AAV infection rate (Fig. 6B) and detected the fusion of gRNA1-2 editing sites in hippocampi infected with AAV-gRNA1-2-CamKII-EGFP, but not AAV-CamKII-EGFP control (Fig. 6C). Like our editing site fusion in the primary cortical cultures, the in vivo fusion took place at the same Cas9 cutting sites (Fig. 6D). Remarkably, AAV expressing gRNA1-2 significantly decreased the percentage of EGFP-positive neurons containing the sense and antisense RNA foci and the numbers of RNA foci in the EGFP-positive neurons, compared to the AAV-CamKII-EGFP controls (Fig. 6E-G). Therefore, our results indicate that our dual-gRNA approach is able to remove the repeat expansion and correct the repeat expansion-generated pathology in vivo.

## Discussion

Here we provide a prove-of-concept design that can correct *C9ORF72* repeat expansion in a ‘cutting-deletion-fusion’ manner, which theoretically corrects the repeat-generated RNA and protein abnormalities at the same time. The sequence low complexity feature of *C9ORF72* repeat site and its surrounding DNA makes it difficult to achieve the cutting accuracy without disturbing *C9ORF72* transcript integrity. Using computational and experimental approaches, we demonstrate that a dual-gRNA outside of the DNA low complexity region have limited off-target effect by removing *C9ORF72* repeat site together with the non-coding exon 1b. In addition, the dual gRNAs do not significantly alter C9ORF72 protein level at least in the cell lines we examined. More importantly, the dual gRNAs remove the repeat expansion and the repeat-generated RNA foci in mouse neurons and brains carrying patient repeat expansion. Therefore, our prototype research in identification of the dual-gRNA may provide therapeutic hope and value for ALS and FTD patients carrying the repeat expansion.

Currently, preclinical treatments for ALS/FTD caused by *C9ORF72* repeat expansion are mainly focused on its abnormal RNA, including antisense oligonucleotide (ASO)-based target RNA silencing, microRNA-based silencing, dCas9-based transcription inhibition, and dCas9-based target RNA editing^11,17,18,49–52^. Those approaches are able to correct RNA foci formed by RNAs transcribed from the GGGGCC repeats. However, the toxic DPR proteins are translated by RAN translation from both sense and antisense repeat RNAs^21,23,25^. It is important for those RNA targeting approaches to consider separate design for targeting both antisense and sense RNAs. Recently, selective reduction of repeat RNA transcription has been emerged as an approach to decrease both antisense and sense RNAs by simply manipulating a single transcription factor^53^. However, so far, no available approach is able to treat all three types of toxicities caused by the GGGGCC repeats at DNA, RNA, and protein levels by a simple one-time treatment.

‘Cutting-deletion-fusion’ mediated by a pair of gRNAs, which removes the DNA sequences between the editing sites, has been shown to successfully rescue the mutant phenotypes in vivo^34–36^. The intronic location of *C9ORF72* repeat expansion makes it an ideal target for this ‘cutting-deletion-fusion’ correction mediated by two gRNAs. First, because of the editing sites located in non-coding region of *C9ORF72*, the editing will not generate out-of-frame mutation. Second, because of production of both RNA foci and DPR proteins dependent on the repeated DNA, the removal of repeated DNA by ‘cutting-deletion-fusion’ theoretically will correct the repeat-generated RNA and protein toxicities at the same time. Indeed, in the cultured neurons and mouse brains expressing patient repeat expansion, we removed the repeated DNA and corrected RNA foci simultaneously (Figs. 5, 6). Although dual-gRNA approach has been employed to excise the *C9ORF72* repeats in patient-derived iPSC^54–56^, the computational and experimental assessment of off target effects of these gRNAs and removal of repeat expansion in brain by these gRNAs have not been achieved so far.

Off-target effect of CRISPR/Cas9 is a major concern for its clinical application. However, using rational online in silico tools^43,44,47^, scientists are able to limit the off-target events^40,57,58^. The 35-bp DNA sequences between *C9ORF72* repeat site and the exon 1b are low complexity, which increases off-target risks by CRISPR/Cas9. Indeed, we employed GUIDE-seq, a cell-based super sensitive off-target detector^40^, to reveal high off-target rate of gRNAb (38.9%), which is consistent with our in silico prediction (Supplementary Table 2). In addition, unlike off-target sites of our gRNA1 and gRNA3 in intergenic regions and introns (Fig. 2), which may have less impacts on genomic DNA function, the majority of gRNAb off-target sites are in the functional regions, including 37.5% in P-TSS, 16.7% in 5’UTR, and 8.3 in coding exons (Fig. 2F). Given that gRNAb is the best candidate we can find in that 35bps (Fig. 1B, C), it is nearly impossible to select high quality gRNA in that 35bps but not bothering the exon 1b. In contrast, our dual-gRNA design has much less off-target effect (gRNA1, 0.2%; gRNA2, undetectable), and even the two reliable off target sites (Off-T1 and Off-T2) of gRNA1 identified by GUIDE-seq were not confirmed by our targeted deep sequencing by at least 2.0 × 10^5^ coverage (Supplementary Fig. 5). Newly-developed an in vitro screen for identifying genome-wide off-target sites could be applied for further interrogation of off-target events of our gRNA1-2^57,59^. In addition, improved CRISPR/Cas9 systems could be applied in the future application to further increase accuracy of CRISPR/Cas9-mediated editing^58,60^.

Although our design removes *C9ORF72* repeat site together with the noncoding exon 1b, this manipulation has limited effect on C9ORF72 protein level at least in HEK293, U251, and SH-SY5Y cells we examined (Supplementary Fig. 5). One out of three human *C9ORF72* transcript variants contains exon 1b, expression which is significantly impaired by the repeat expansion^2^. The C9ORF72 N-terminal antibody we used here detects C9ORF72 protein that could be encoded by both exon 1a- and exon 1b-containing transcripts. It will be intriguing to know whether expression level of individual *C9ORF72* transcript variants is affected when the repeat site and exon 1b are removed by our dual-gRNA design. Although we included 3 single cell clones in which 2 alleles of repeat site and exon 1b were removed by gRNA1-2, none of them showed C9ORF72 protein drop compared to non-editing controls (Fig. 3C-G). Expression of *C9ORF72* differs from cell to cell with much higher expression in patient-derived motor neurons^18^. Further studies are needed to carefully examine the expression level of *C9ORF72* transcript variants and C9ORF72 protein when the repeat expansion and exon 1b are removed by our gRNAs in those patient-derived motor neurons. Patients showed lower C9ORF72 expression while the proportions of the three transcript variants (V1, V2, and V3) are similar in cerebellum and frontal cortex between patients and controls^61^. Though we did not show obvious C9ORF72 protein change by removing exon 1b in HEK293, U251, and SH-SY5Y cells, the removal may have effects on these transcript variant regulation, localization, and balance. Completely removing G4C2 repeats may have some potential ramifications considering that a normal person carries ~ 3–24 G4C2 repeats. The physiological functions of short G4C2 repeats in *C9ORF72* expression and functions need to be further studied. High fusion rate at two distal editing sites is important for this ‘cutting-deletion-fusion’ manipulation^34–36^. Using the Southern blot, we estimated that our gRNA1-2 fusion rate is 49.5 ± 5.8% (Fig. 4C), at least in cells expressing both Cas9 and gRNA1-2. Because the production of RNA foci depends on the repeated DNA expansion, correction rate of RNA foci by our dual gRNAs reaches ~ 50% in cultured neurons derived from Cas9/ + ;C9-Tg mouse, which carries ~ 100–1000 copies of G4C2 repeats from the patient and constitutively expresses spCas9 (Fig. 5D, E), suggesting ~ 50% fusion in these neurons. Therefore, our results suggested that if both the dual gRNAs and spCas9 can be delivered successfully into a cell/neuron, the fusion rate or removal rate of repeats is most likely to reach ~ 50%. However, *C9Orf72* repeat expansion is autosomal dominant in the patient. It will be intriguing to examine the fusion rate by our dual-gRNA in the patient-derived cells, like patient-derived iPSC. In the future AAV-based therapeutic setting, the real fusion rate will be taken account of AAV delivery rate of gRNAs and spCas9 into diseased neurons. The high fusion efficiency could have more therapeutic value for non-dividing cell like neuron, because daughter cell inherits genetic modification from its parental dividing cell after Cas9 editing, if the modification is beneficial for cell viability, which will lead to increased editing efficiency^34–36^.

For future clinical application, shorter Cas9 from *Staphylococcus aureus* (SaCas9) could be employed^62^, which allows to pack two gRNAs and SaCas9 in a single AAV particle to ensure delivery of both SaCas9 and gRNAs into a target cell. However, SaCas9 also has its own limitations. Compared to the simple SpCas9 PAM sequences (NGG), SaCas9 has complex PAM sequences (NNGRRT), which limits the options of SaCas9-based gRNA selection especially in a short genomic region^62,63^, like the region (from *C9ORF72* exon1a to the repeat site, 162bps) for upstream gRNA selection in our case. For excision of long DNA sequences, like the large repeated DNA in *C9ORF72* in our case, Bengtsson et al. demonstrated that dual-vector, in which the SpCas9 and gRNAs were packed separately into two AAVs, had much higher editing efficiency than that of single-vector in which the SaCas9 and gRNAs were packed together into one AAV in treatment of DMD^64^. In addition, separate packing also allows researchers to adjust the amount of AAV-SpCas9 and AAV-gRNAs, the proportion of which has been demonstrated to have profound effect on target sequence editing efficiency in vivo^65^. Using separate packing, ones even can increase the number of gRNAs they select, for example in our case we can include more than two pairs of gRNAs flanking the repeat site, which has been proven to significantly improve the removal rate of large piece of genomic DNA^66^. Because pre-clinical studies have shown that the separate packing approach achieved the efficacy of large DNA fragment removal in treatment of DMD and Huntington’s disease in vivo^36,67^, we reason that this approach may have high successful rate in treatment of large *C9ORF72* repeats in future pre-clinical study.

With the advance of other delivery means in vivo, like lipid-based delivery of Cas9-gRNA complexes^68^, we may have more choices in the future to deliver the SpCas9 and gRNA(s) together more efficiently in vivo. Because high prevalence of exposure to *S. pyogenes* and *S. aureus* in human population, immunogenicity of SpCas9 and SaCas9 is a potential obstacle to its clinical application^32^. Identification and characterization of novel Cas9 orthologues with low immunogenicity could be a way to overcome the obstacle. Given that CNS is immune-privileged and local delivery of AAV-based Cas9/gRNAs through intrathecal injection is restricted in CNS^32,69^, the whole body Cas9 immunogenicity could be less concerned. Controllable expression of Cas9 protein could be also beneficial to avoid the immunogenicity during future clinical application.

## Supplementary Information

Supplementary Information.
