# Supplementary material for: Dual-gRNA approach with limited off-target effect corrects C9ORF72 repeat expansion in vivo
Source: Sci Rep. 2022 Apr 5;12:5672. doi: 10.1038/s41598-022-07746-8 (PMC8983752; doi:10.1038/s41598-022-07746-8)
Supplement: Supplementary file 1 — Supplementary Information. [file 41598_2022_7746_MOESM1_ESM.docx]

**Dual-gRNA approach with limited off-target effect corrects *C9ORF72* repeat expansion *in vivo***

Xuejiao Piao^1, 2, 3, 4, 5^, Dawei Meng^1, 2, 3, 4, 5^, Xue Zhang^1, 3, 4, 5^, Qiang Song^1, 4^, Hailong Lv^1, 2, 3, 4^, and Yichang Jia^1, 3, 4, #^

^1^ School of Medicine, Medical Science Building, Room D204, Tsinghua University, Beijing, China, 100084.

^2^ School of Life Sciences, Tsinghua University

^3^ Peking-Tsinghua Joint Center for Life Sciences.

^4^ IDG/McGovern Institute for Brain Research at Tsinghua.

^5^ These authors contributed equally to this work.

# Corresponding author

Please address correspondence to:

Yichang Jia, Ph.D.

School of Medicine, Medical Science Building, Room D204, Tsinghua University, Beijing, 100084, P. R. China

Tel: 86-10-62781045

Email: [yichangjia@tsinghua.edu.cn](mailto:yichangjia@tsinghua.edu.cn)

**
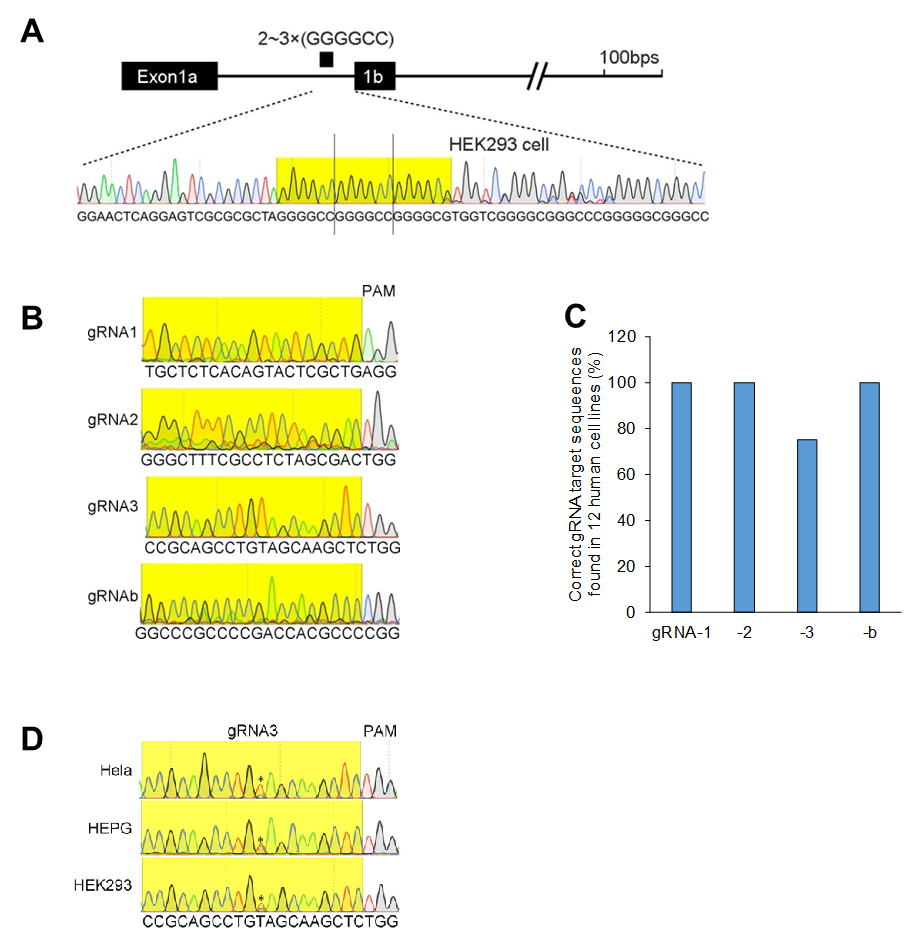
**

**Supplementary Fig. 1. The existence of the repeat site and our gRNA target sequences in human cell lines we examined.**

(**A**) In HEK293 cell, 2~3 GGGGCC repeats in the *C9ORF72* intron. The sequence highlighted indicates the GGGGCC repeats. (**B**) The target sequences of gRNA1, gRNA2, gRNA3, and gRNAb were detected by Sanger sequencing in HEK293 cell. The sequences highlighted indicate gRNA target sequences. (**C** and **D**) No genetic variance was detected in gRNA1, 2, and b target sequences in the 12 human cell lines we examined, including BJ, HaCaT, HEK293, Hela, HEPG, Huh7.5, HUVEC, IMR-90, MRC-5, OS-732, SH-SY5Y, and U2OS cells. A SNP (asterisk in **D**) in gRNA3 target sequences was detected in 3 out of these 12 cell lines. The sequences highlighted indicate gRNA target sequences.

**Supplementary Fig. 2. Lentiviral dual-gRNA delivery system.**

The system modified from previous studies (PMID: 26493208 and 25075903). The dual gRNAs are driven by U6 and H1 promoters, separately. In control, the sequences downstream of U6 promoter are spacer sequences (PMID: 25075903).

**
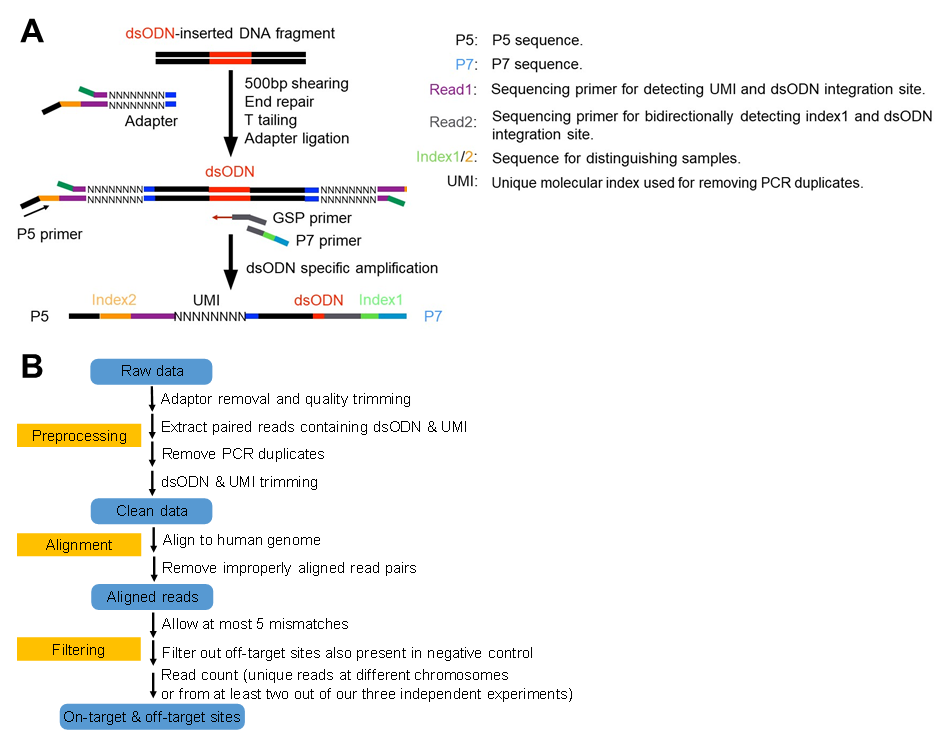
**

**Supplementary Fig. 3. The pipeline of GUIDE-seq.**  (**A**) Schematic overview of GUIDE-seq (PMID: 24584096) with slight modification. HEK293 cells constructively expressing Cas9 were transfected with dual gRNAs together with a blunt double-stranded oligodeoxynucleotide (dsODN). Genomic DNA containing oligo tag integration sites were sheared into 500bps in length. After adaptor ligation, the resulting products were amplified by oligo tag specific amplification and applied for next-generation sequencing. UMI (unique molecular index) was used for removing the PCR duplicates and counting the numbers of unique integration sites. (**B**) The flow of GUIDE-seq data analysis. In brief, the raw data went through three major steps to achieve the reliable dsODN integration sites. We included off-target events that were supported by unique reads at different chromosomes or from at least two out of our three independent experiments.

**
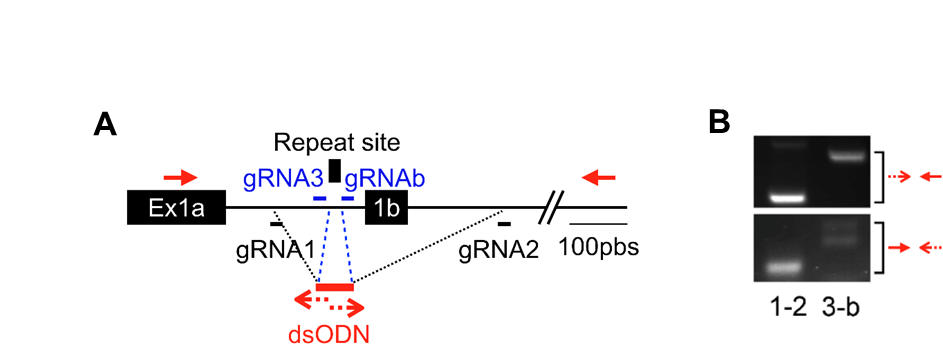
**

**Supplementary Fig. 4. Validation of on-target integration of dsODN.** (**A**) The primer locations for the validation. (**B**) The on-target integration sites were validated by genomic DNA PCR with the indicated primers (**A**) in HEK293 cells constructively expressing Cas9 and transfected with the gRNA1-2 or gRNA3-b together with dsODN.

**
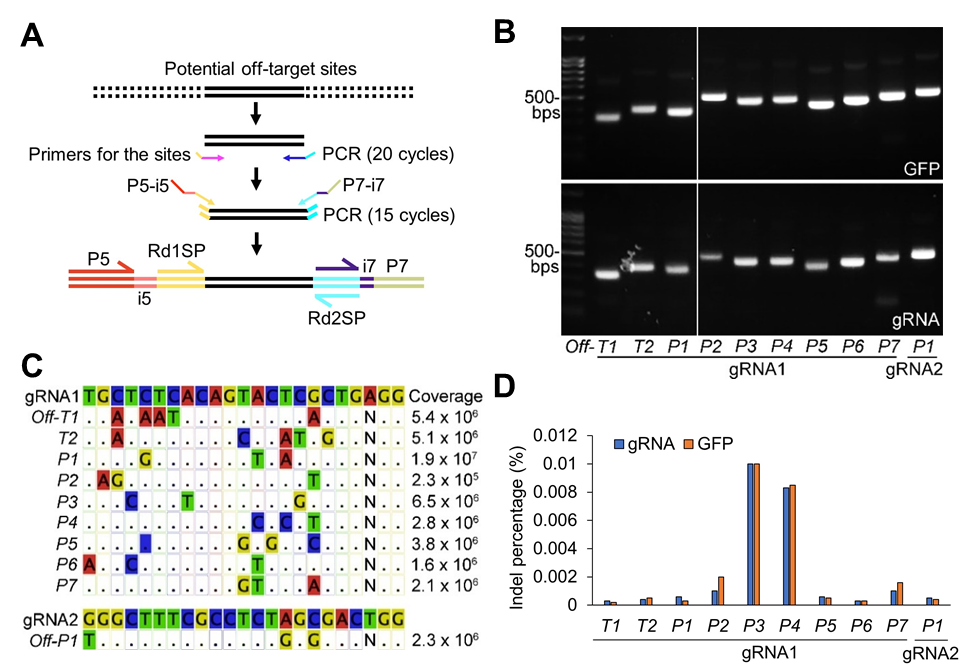
**

**Supplementary Fig. 5. Confirmation of off-targets of gRNA1 and gRNA2.**  (**A**) Schematic overview of our off-target confirmation by targeted deep sequencing. Index 5, i5; index 7, i7; Rd1SP, Read 1 sequencing primer; Rd2SP, Read 2 sequencing primer (Illumina). (**B**) HEK293 cells constitutively expressing Cas9 were transfected with GFP, gRNA1, or gRNA2. The PCR amplicons of off-target sites of gRNA1 and gRNA2 identified by GUIDE-seq (**Supplementary Table 2**). (**C**) Targeted deep sequencing coverage of individual off-target sites. (**D**) Percentage of off-target site indels in HEK293 cells constructively expressing Cas9 and transfected with gRNA1, gRNA2 or GFP.

**
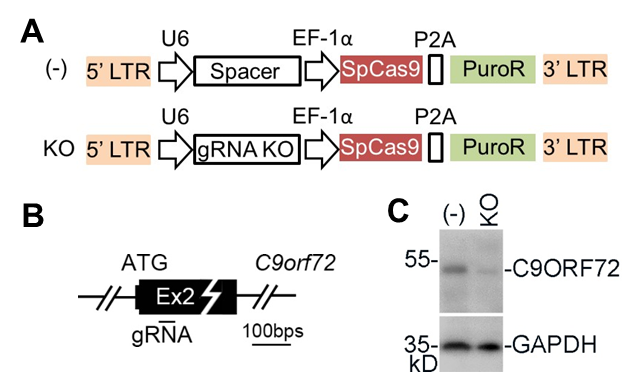
**

**Supplementary Fig. 6. Validation of a C9ORF72 antibody.** (**A**) For C9ORF72 antibody validation, we employed a lentiviral Crispr/Cas9-based gene knock-out (KO) system (PMID, 25075903). (**B**) The genome location of gRNA for *C9ORF72* KO. (**C**) The protein lysates from the control (-) or HEK293 cells infected with lentiviral particles expressing the KO gRNA were blotted with the C9ORF72 antibody.

**
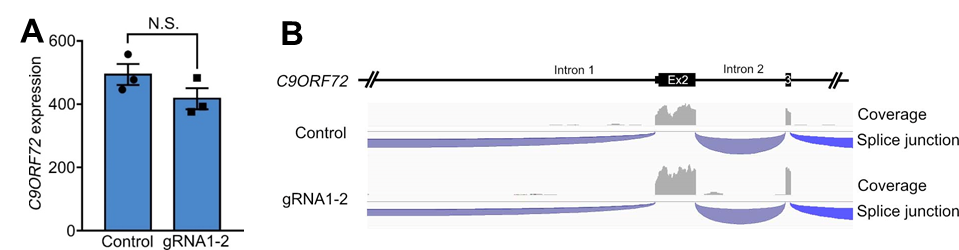
**

**Supplementary Fig. 7. *C9ORF72* expression in HEK293 cells treated with dual-gRNA.** (**A**) *C9ORF72* expression in HEk293 cells treated with the dual-gRNA detected by RNA-seq. The normalized read counts were quantified by DESeq2 (n=3). N.S., no statistical significance. (**B**) The splice junctions nearby the first coding exon (exon 2) visualized by Integrative Genomics Viewer (IGV).

**
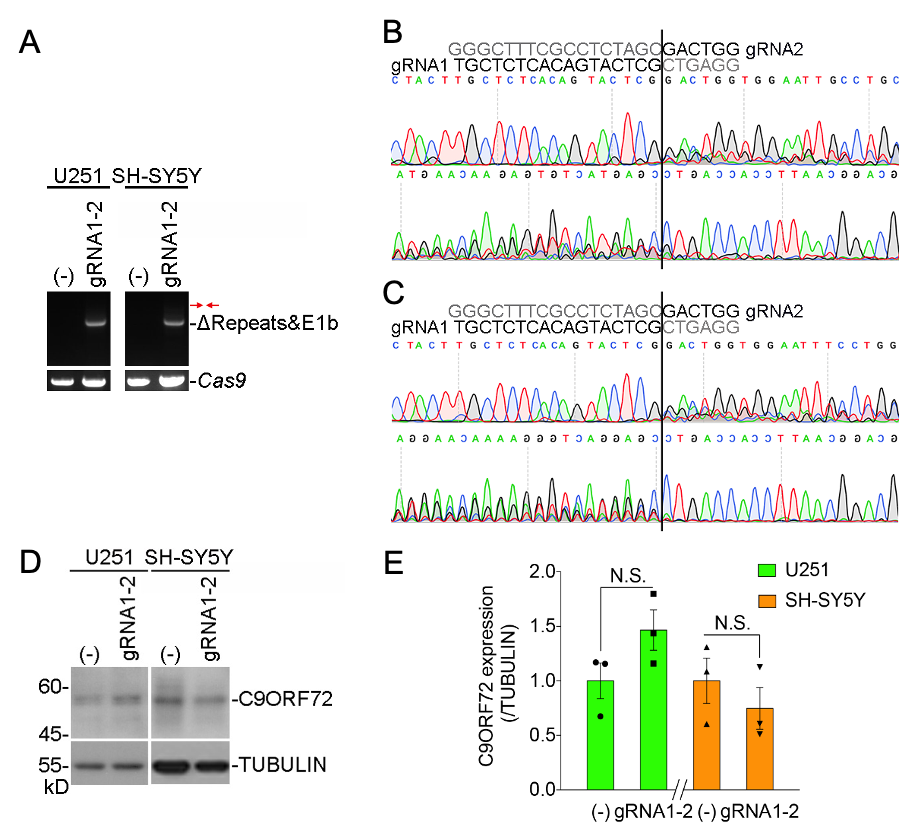
**

**Supplementary Fig. 8. Deletion of repeat site and exon 1b by gRNA1-2 on C9ORF72 protein expression in U251 and SH-SY5Y cells.** (**A**) Genomic DNA PCR for detecting the removal of repeat site and exon 1b (ΔRepeats+E1b) by gRNA1-2 in U251 and SH-SY5Y cells, respectively. The cells constitutively expressing Cas9 were infected with the lentiviral control and gRNA1-2, respectively (**Supplementary Fig. 2**). The band of ΔRepeats+E1b appeared in cells infected with gRNA1-2 but not control (-) due to the removal of the GC-rich region. Here we lowered high GC buffer in the PCR condition as shown in **Fig. 1D**. (**B** and **C**) The deletion bands (Δ) shown in (**A**) were applied for both forward and reverse Sanger sequencing. In b, U251; in c, SH-SY5Y. (**D**) Representative image of C9ORF72 immunoblot from U251 and SH-SY5Y cells infected with control (-) or gRNA1-2 lentiviral particles. TUBULIN served as loading control. (**E**) Relative expression of C9ORF72 (normalized to TUBULIN) shown in (**D**). In e, the protein lysates from at least three biological replicates and the values presented as mean ± SEM (n= 3). N.S., no statistical significance (t-test, SPSS).

**
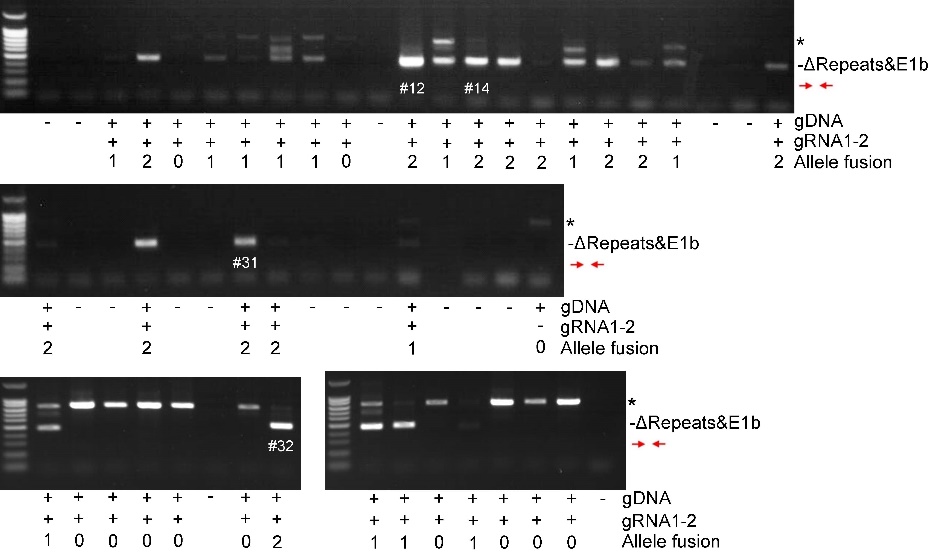
**

**Supplementary Fig. 9. Single cell clonal analysis for fusion rate estimation by our dual-gRNA.** HEK293 cells constitutively expressing Cas9 were infected with our dual-gRNA. After puromycin selection, cells were plated as single clone. Genomic DNA (gDNA) from each clone was extracted and applied for PCR for fusion rate estimation (the primers shown in **Figure 1A**). Of the 38 clones positive for PCR band(s), 13 clones with two allele fusion, 13 with single allele fusion, and 12 without fusion. The calculated fusion rate is (39/76, 51.3%). *, no fusion PCR band; ΔRepeats&E1b, fusion PCR band; -, negative PCR band.

**
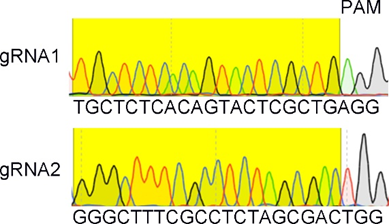
**

**Supplementary Fig. 10. The existence of our gRNA target sequences in *C9ORF72*-BAC transgenic mouse.** The *C9ORF72*-BAC transgenic mouse (C9-Tg) carries the expanded GGGGCC repeats from patient (C9-Tg mouse line 112, PMID: 26637796). The gRNA1 and gRNA2 target sequences were detected in this transgenic mouse by Sanger sequencing. The sequences highlighted indicate gRNA target sequences.

**
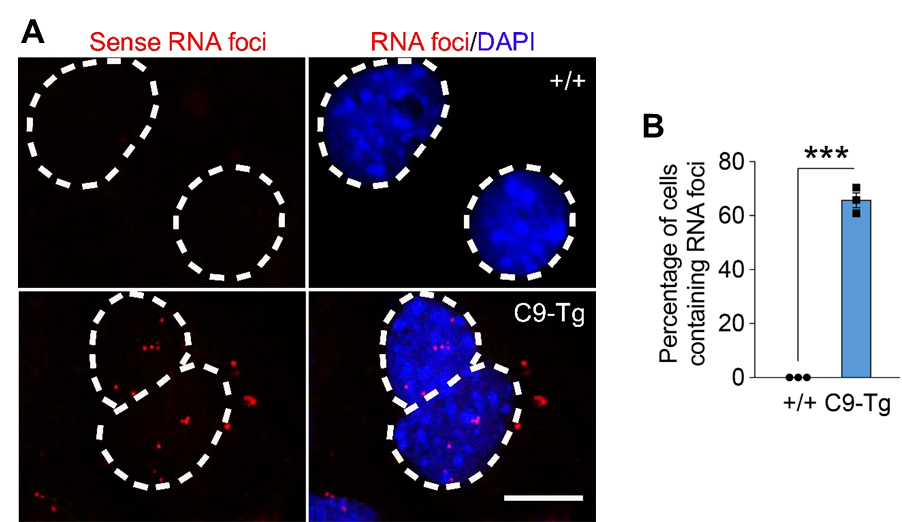
**

**Supplementary Fig. 11. The RNA foci shown in the primary cortical cultures carrying patient repeats.** (**A** and **B**) Sense RNA foci were detected in the primary cortical cultures derived from the *C9ORF72*-BAC transgenic (C9-Tg) mice but not in the wild type (+/+) control. In a, the dotted line circled nuclei of cultured neurons with indicated genotypes. In **B**, the RNA foci counts from three biological replicates and the values are presented as mean ± SEM (n = 3). ****p* <0.001 (t-test, SPSS). Scale bar in (**A**), 10 μm.

**
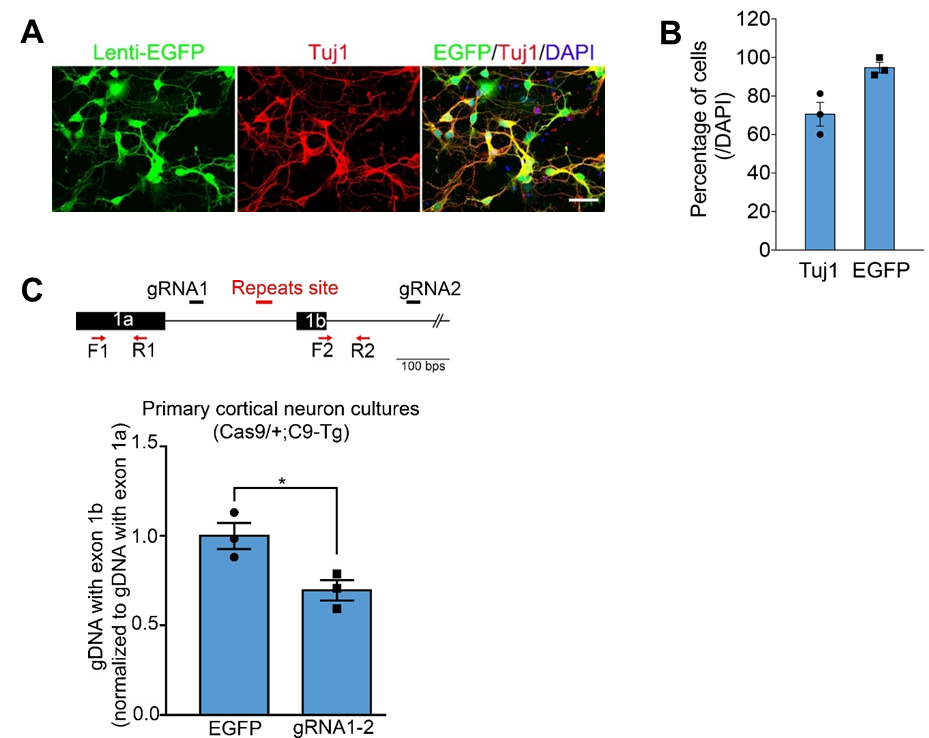
**

**Supplementary Fig. 12. Our lentiviral infection rate in our primary cortical cultures and the estimated fusion rate of *C9ORF72* mutant allele by our dual-gRNA.** (**A**) Representative images of our primary cortical cultures (P2, 4 DIV) isolated from wild type (+/+) mouse. Tuj-1 was used as a neuronal marker. Scale bar, 20 μm. (**B**) The percentage of Tuj-1 and GFP-positive cells in our cultures. (**C**) Quantitative PCR was employed to estimate fusion rate of *C9ORF72* mutant allele by our dual-gRNA. Genomic DNA (gDNA) extracted from Cas9/+;C9-Tg cortical neuron cultures infected with EGFP or our dual-gRNA was used for quantitative PCR. For gDNA with exon 1a, primers: F1 and R1; for gDNA with exon 1b, primers: F2 and R2. The values are presented as mean ± SEM (n = 3). **p* <0.05 (t-test, SPSS).

**
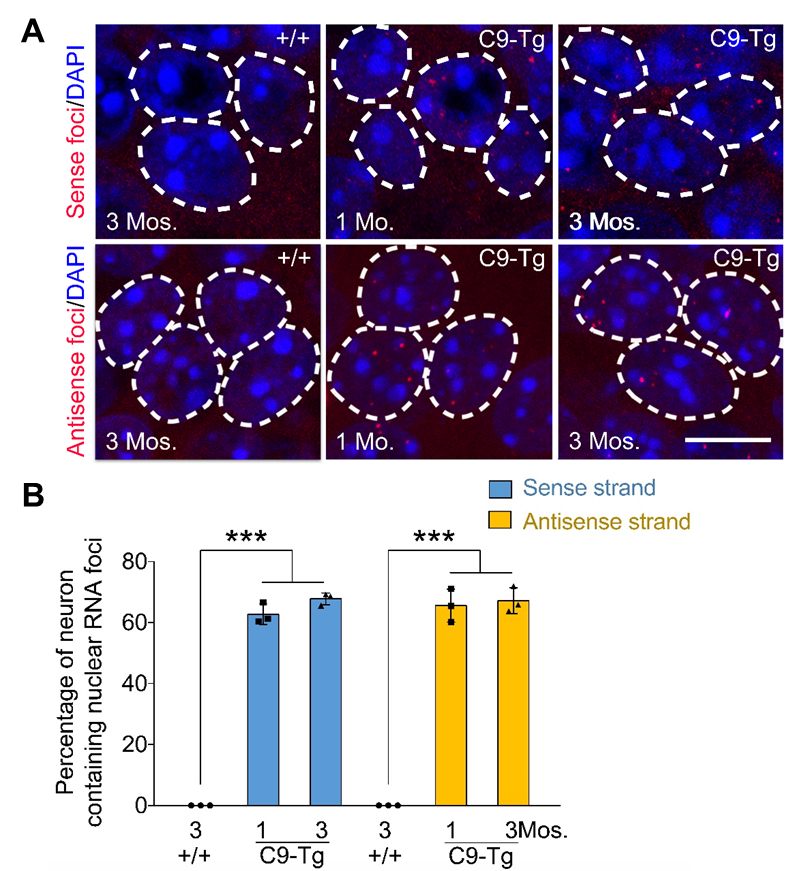
**

**Supplementary Fig. 13. The RNA foci shown in hippocampal CA1 regions in C9-Tg mice**. (**a**) Both sense and antisense RNA foci were detected in C9-Tg hippocampal CA1 neurons at indicated age points. The dotted line circled nuclei of CA1 neurons with indicated genotypes and ages. Mo., month. (**b**) Data summary of sense and antisense RNA foci detected in CA1 neurons shown in (**a**). The values are presented as mean ± SEM (n = 3). ****p* <0.001 (ANOVA, SPSS). In (**a**), scale bar,10 μm.

**
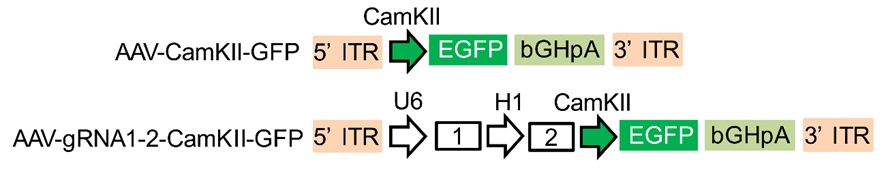
**

**Supplementary Fig. 14. AAV-based dual-gRNA delivery system *in vivo*.** The GFP driven by CamKII promoter is used for tracing the infected regions and neurons *in vivo*.


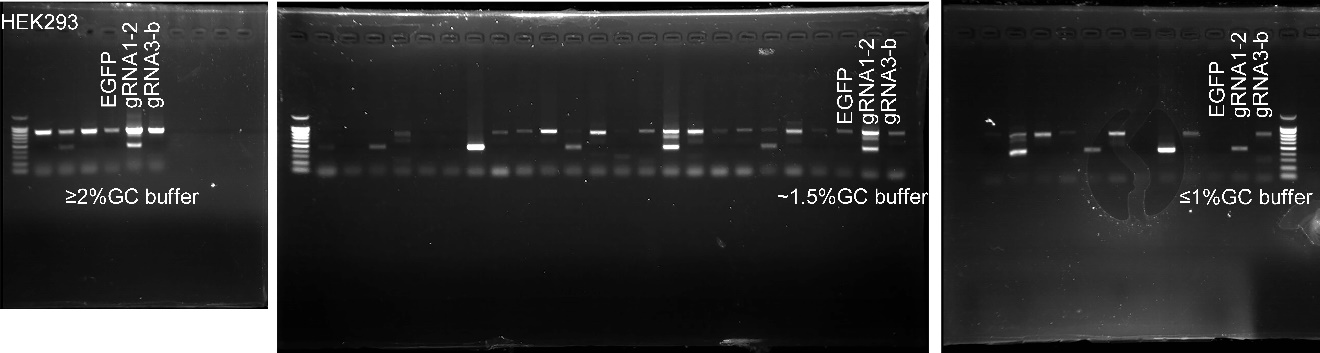


**
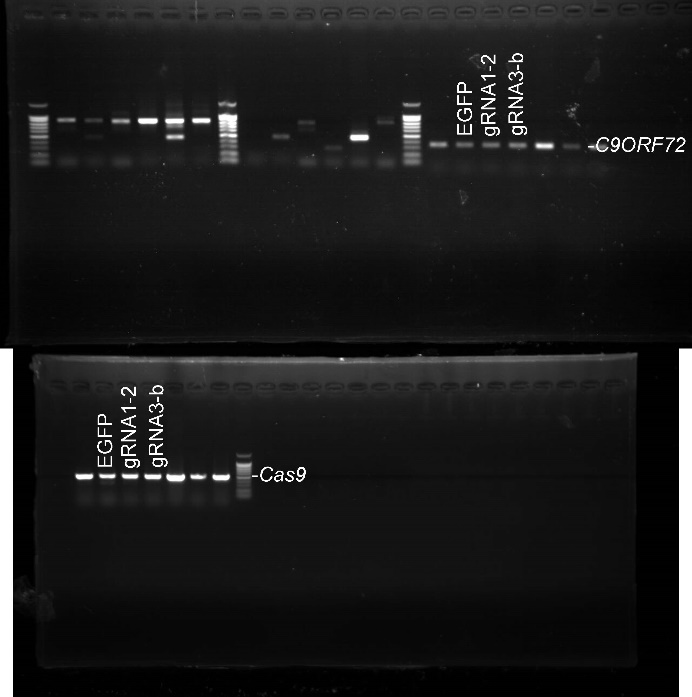
**

**Supplementary Fig. 15. Original uncropped DNA gel for Fig. 1D.**

**
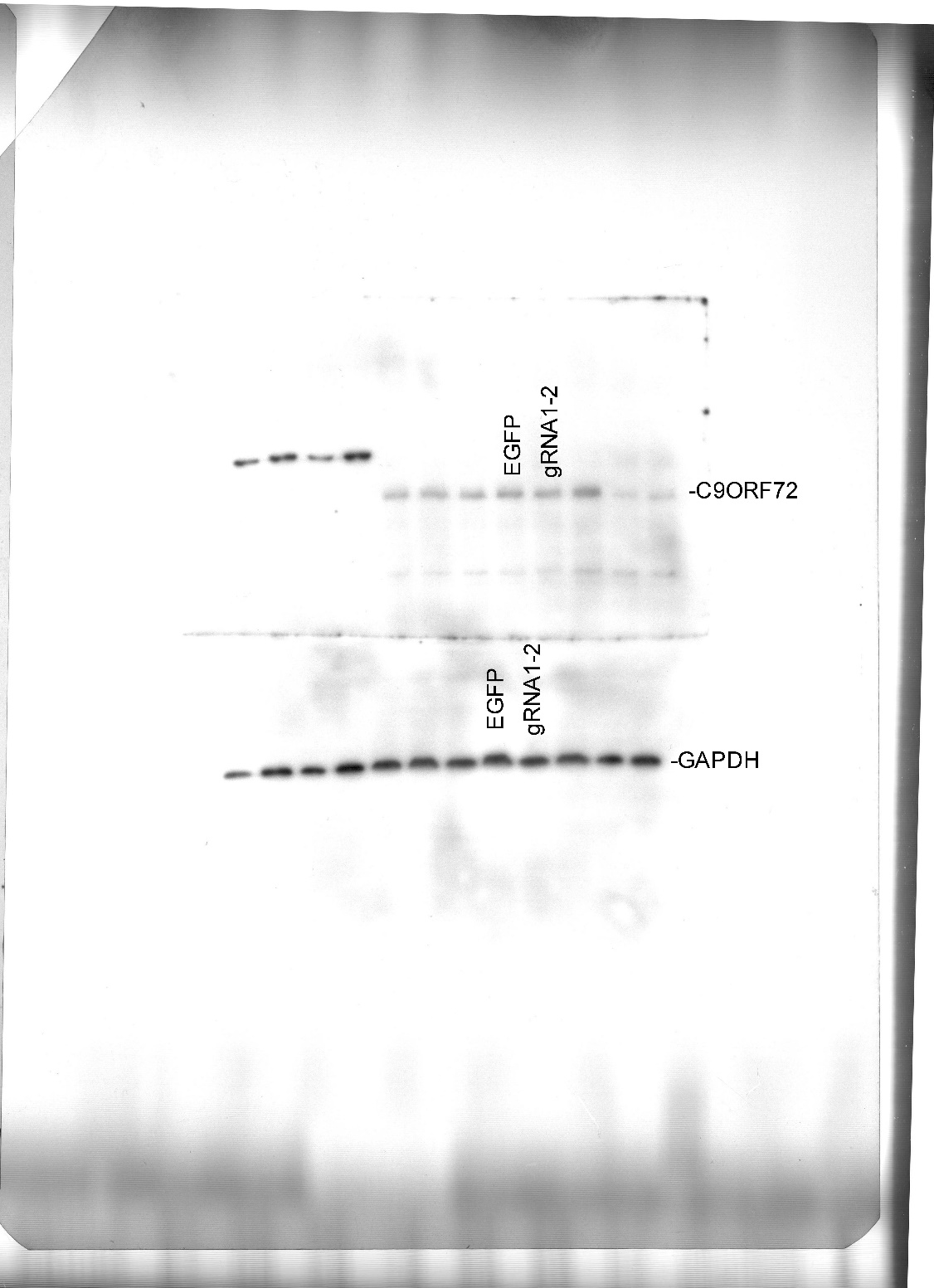
**

**Supplementary Fig. 16. Original uncropped Western blot images for Fig. 3A.**

**
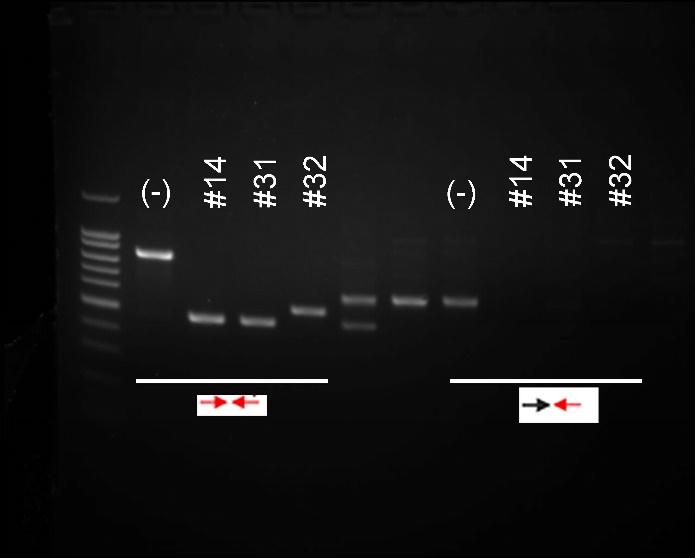
**

**Supplementary Fig. 17. Original uncropped DNA gel for Fig. 3D.**


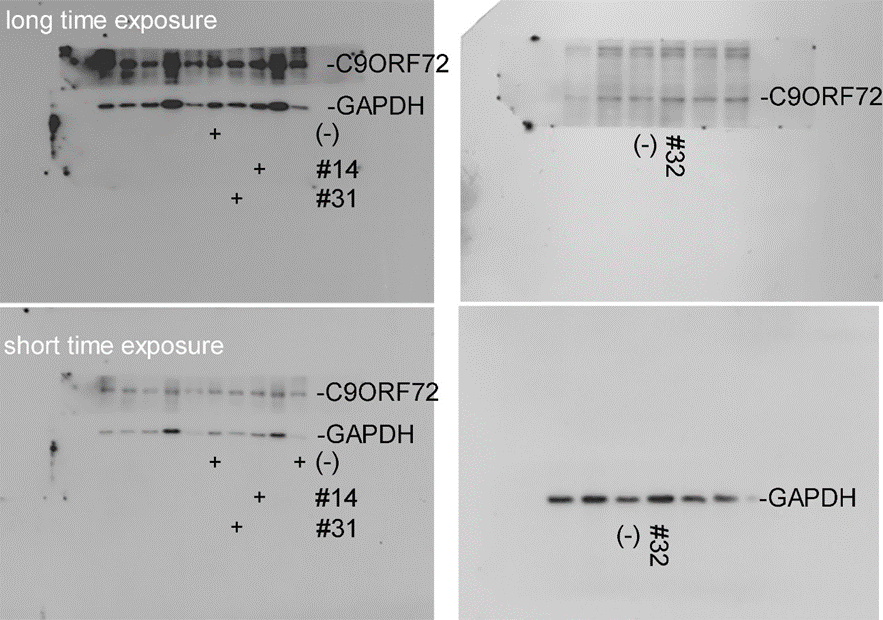
**Supplementary Fig. 18. Original uncropped Western blot images for Fig. 3F.**

**
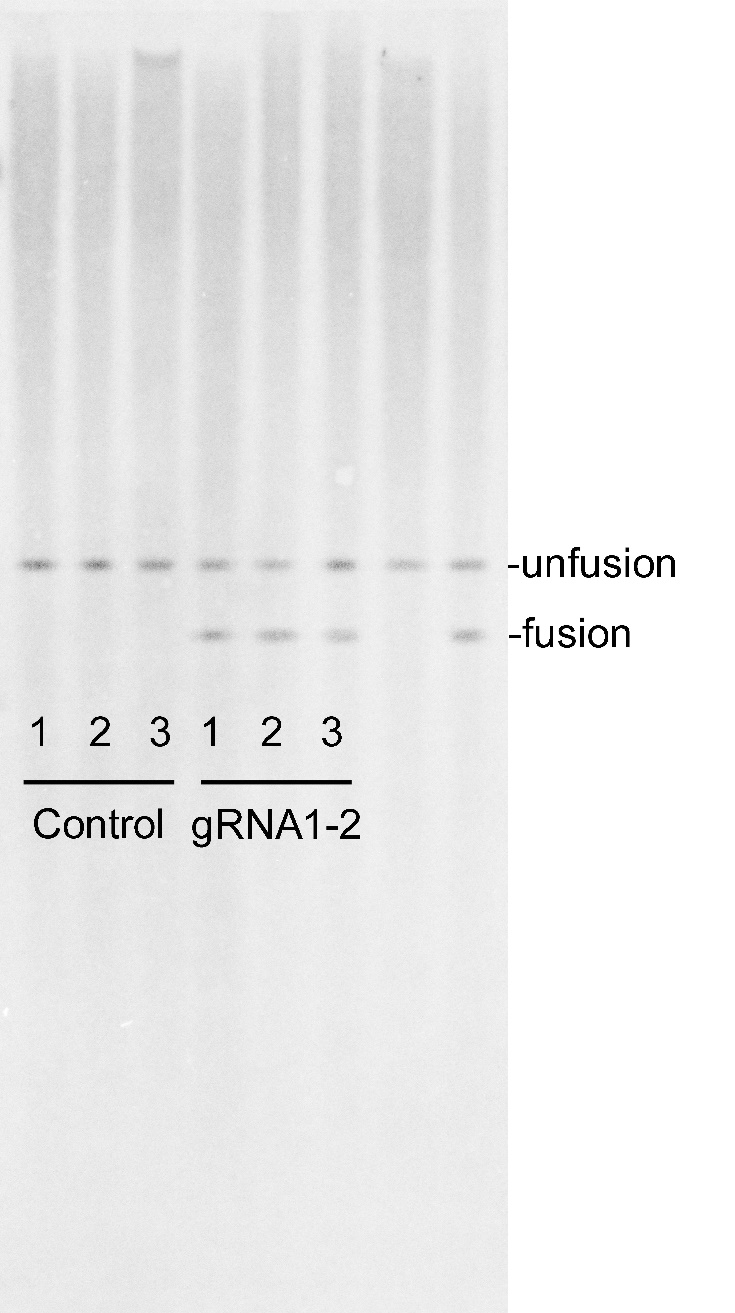
**

**Supplementary Fig. 19. Original uncropped Northern blot images for Fig. 4B.**

**
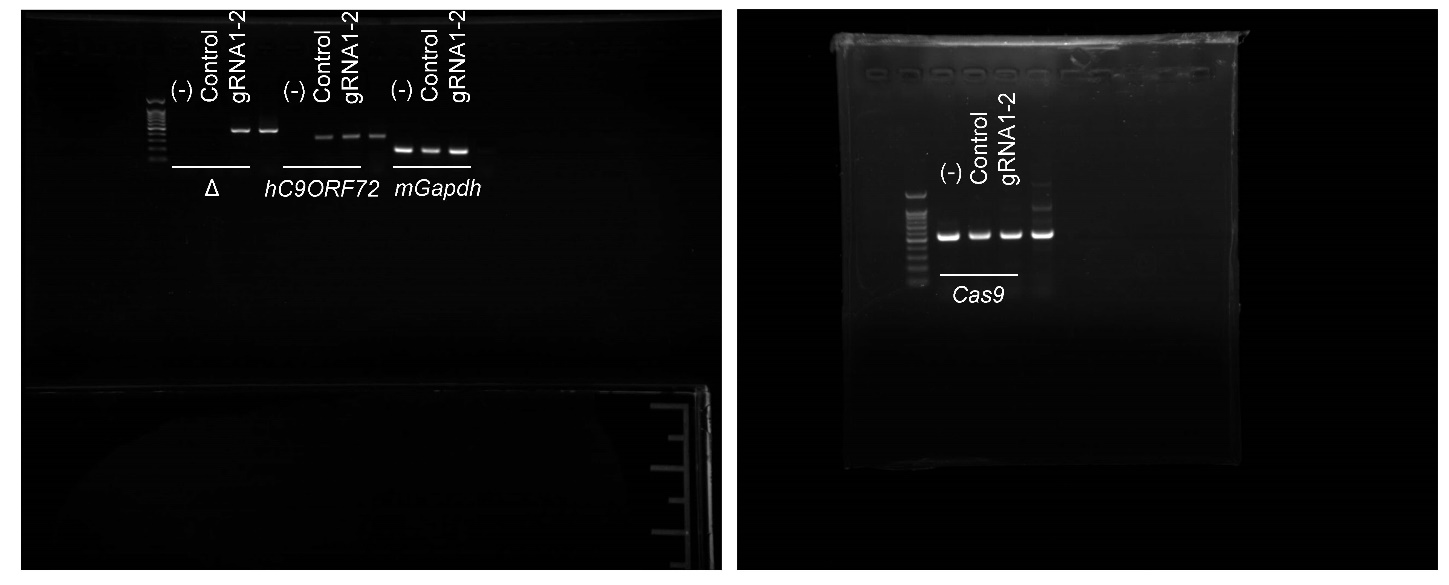
**

**Supplementary Fig. 20. Original uncropped DNA gel for Fig. 5A.**

**
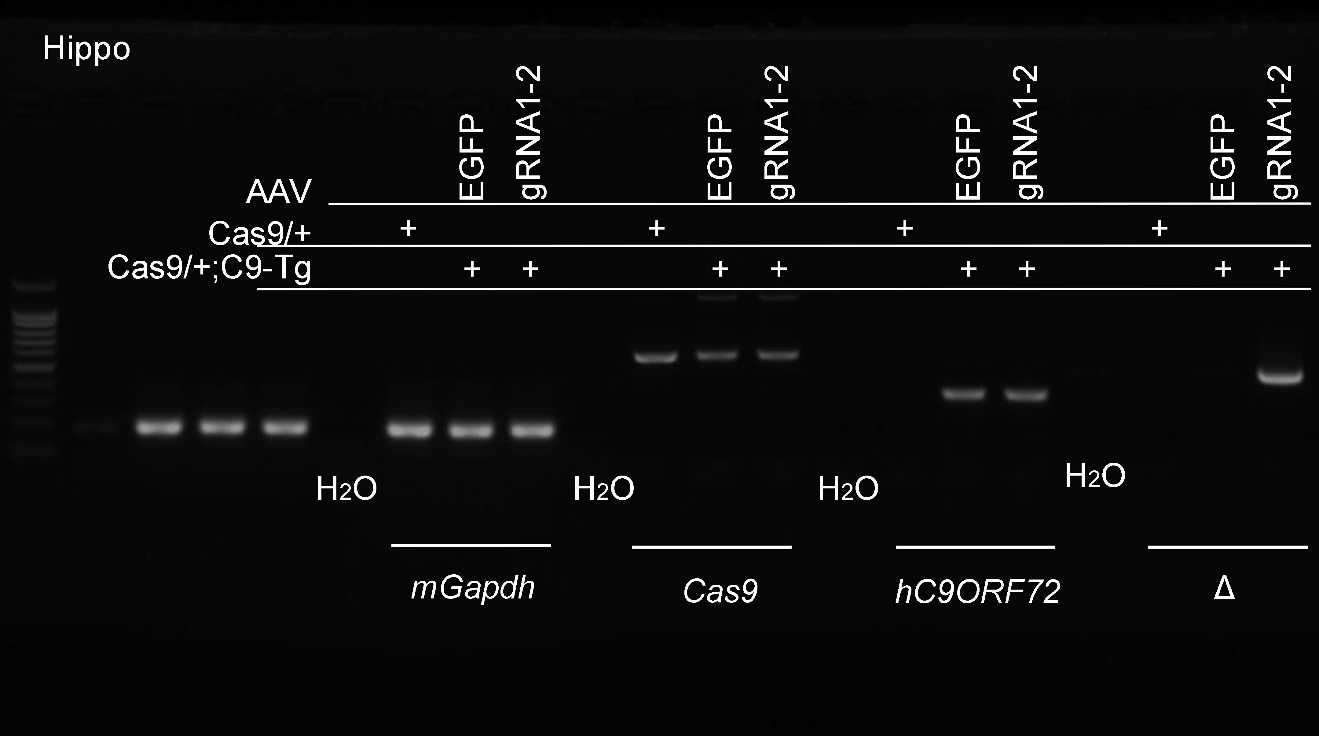
**

**Supplementary Fig. 21. Original uncropped DNA gel for Fig. 6C.**

**
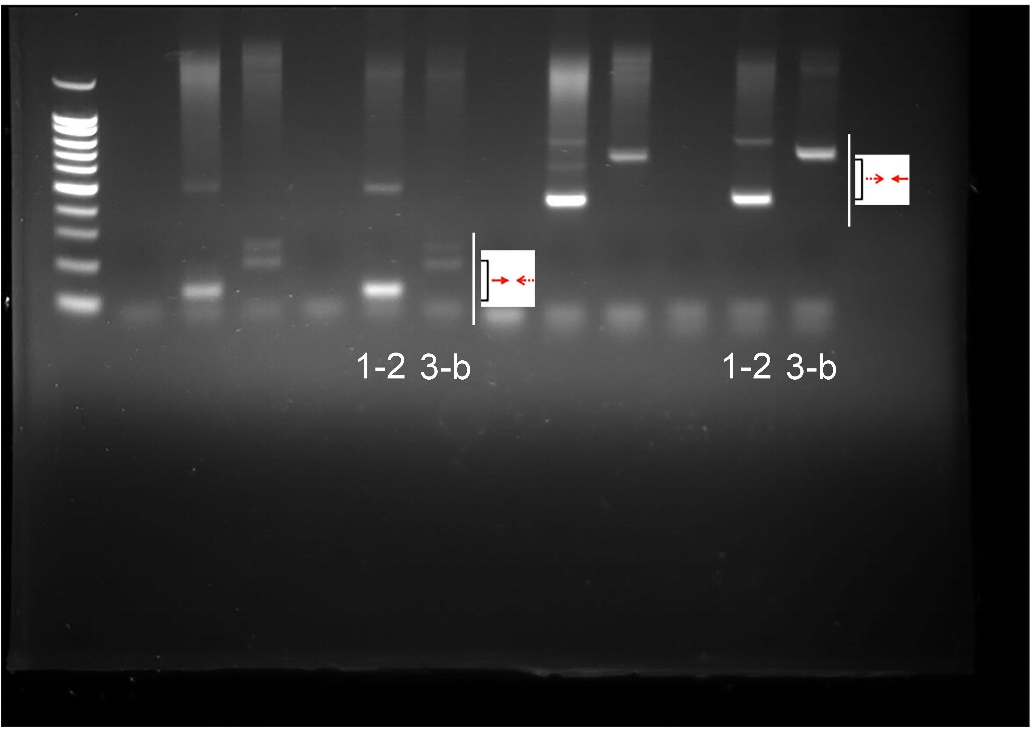
**

**Supplementary Fig. 22. Original uncropped DNA gel for Supplementary Fig. 4B.**

**
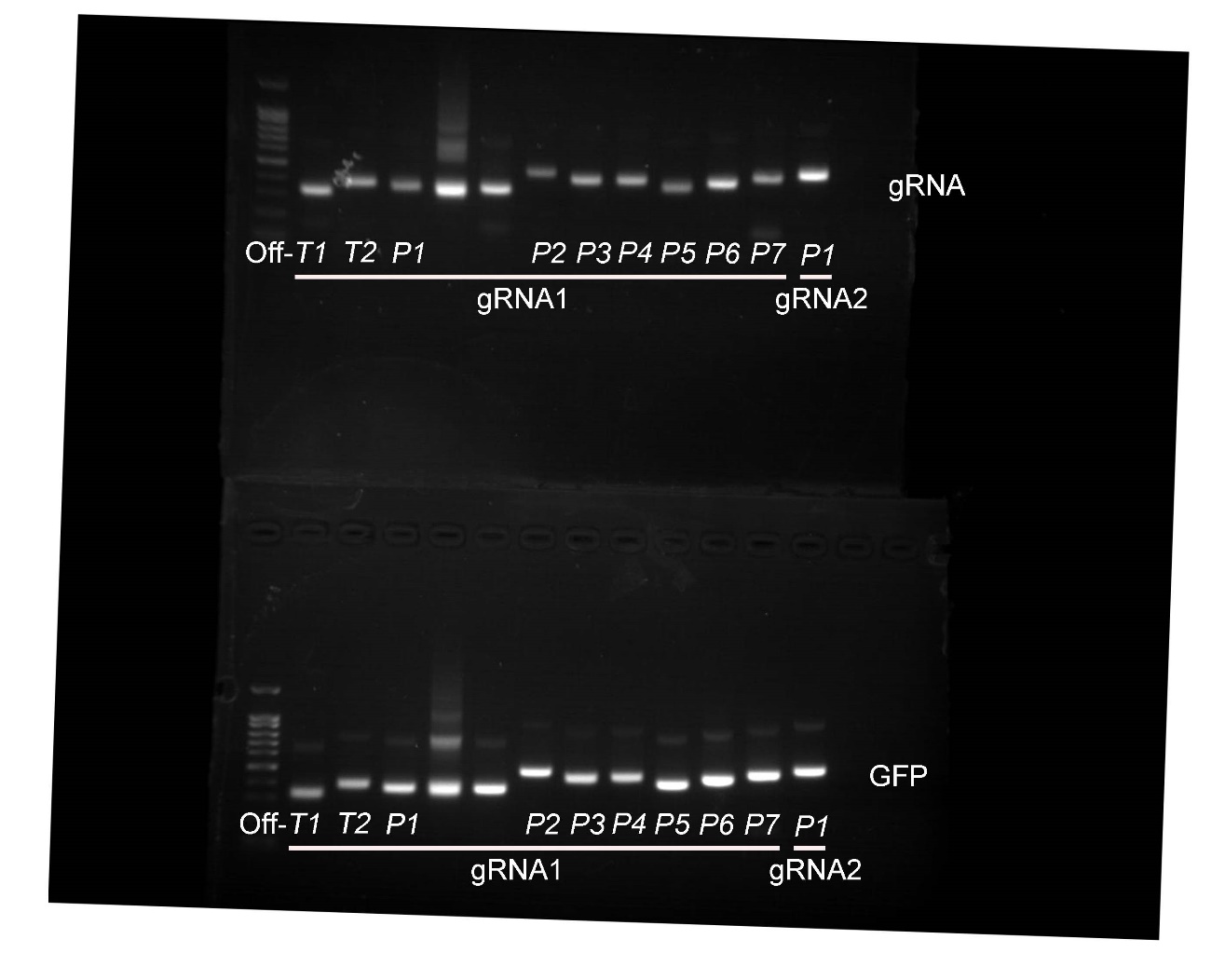
**

**Supplementary Fig. 23. Original uncropped DNA gel for Supplementary Fig. 5B.**

**
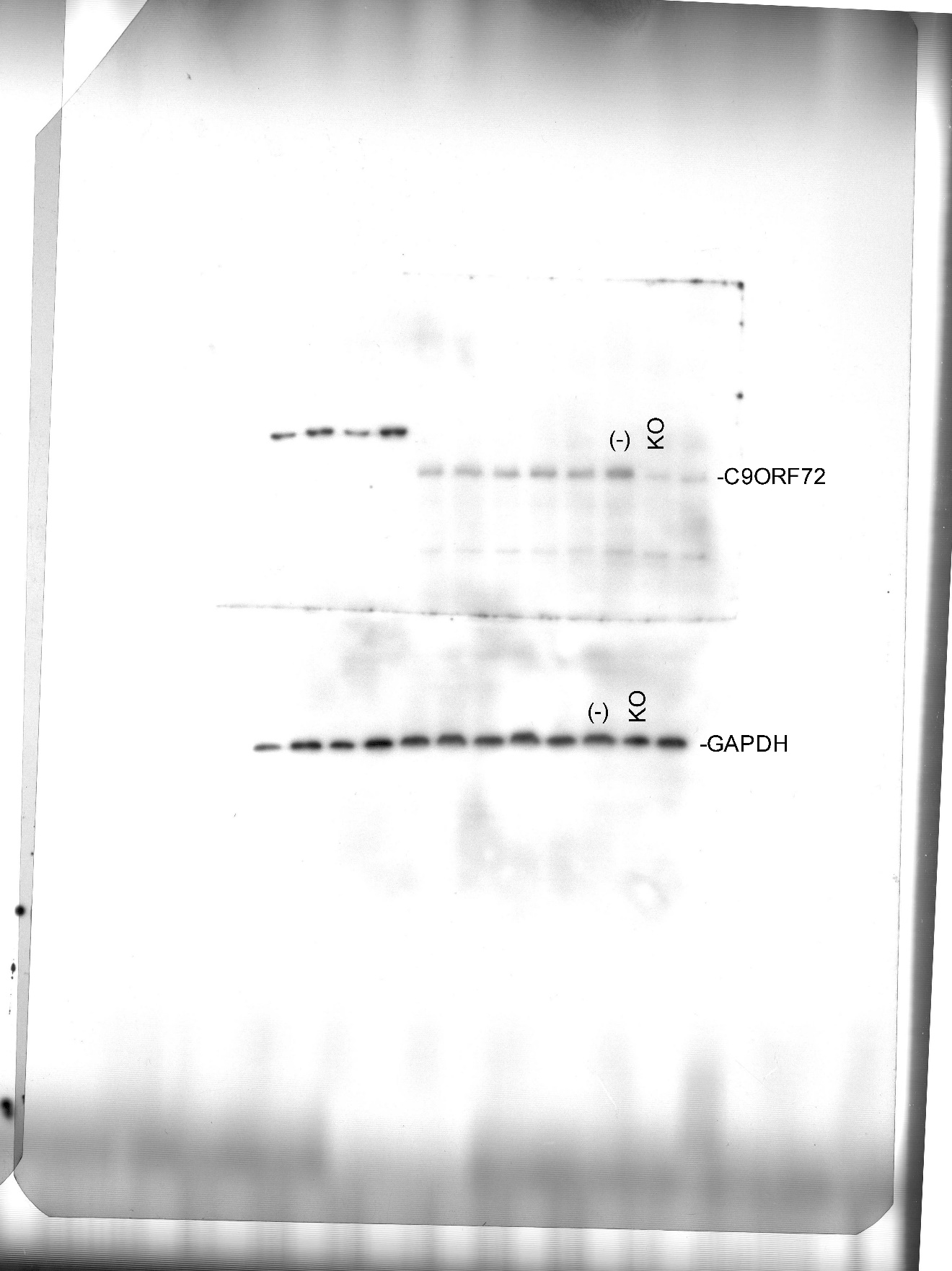
**

**Supplementary Fig. 24. Original uncropped Western blot images for Supplementary Fig. 6C.**

**
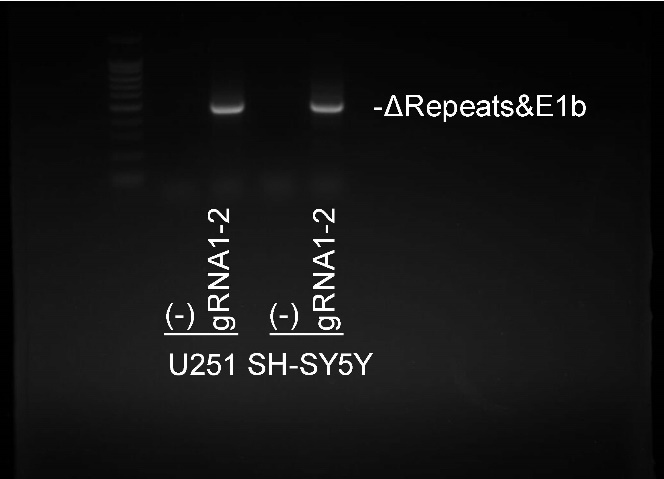
**

**
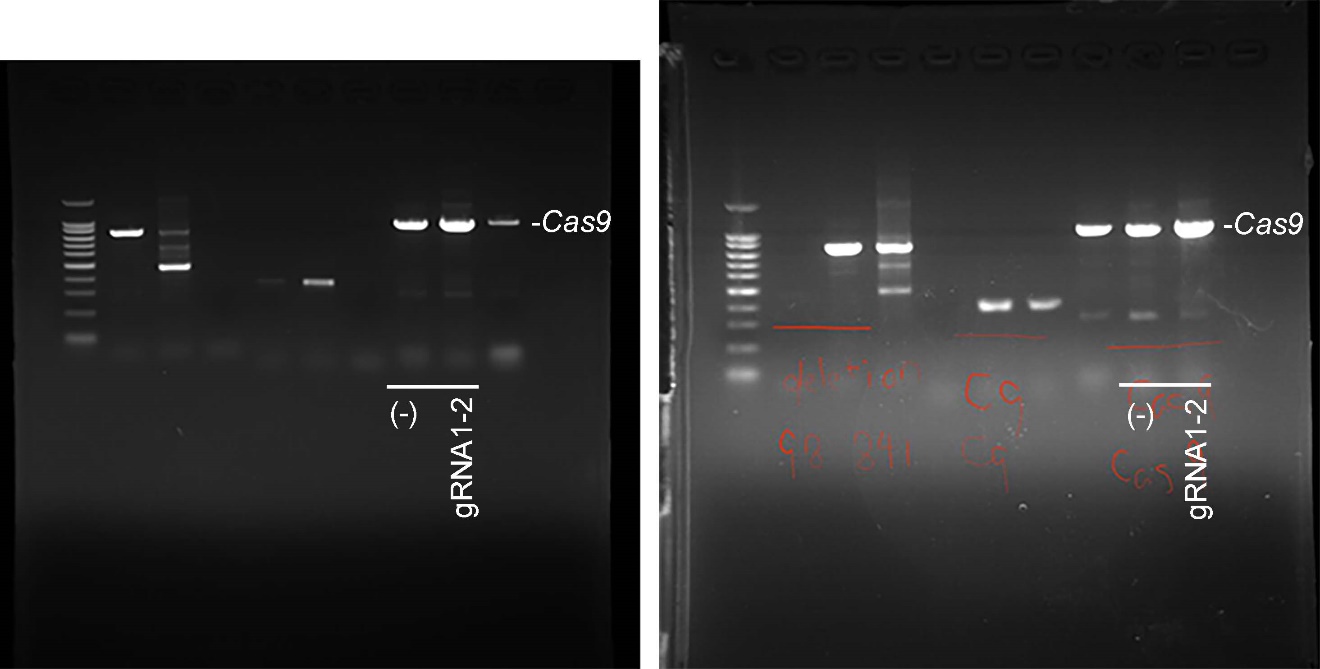
Supplementary Fig. 25. Original uncropped DNA gel for Supplementary Fig. 8A.**

**
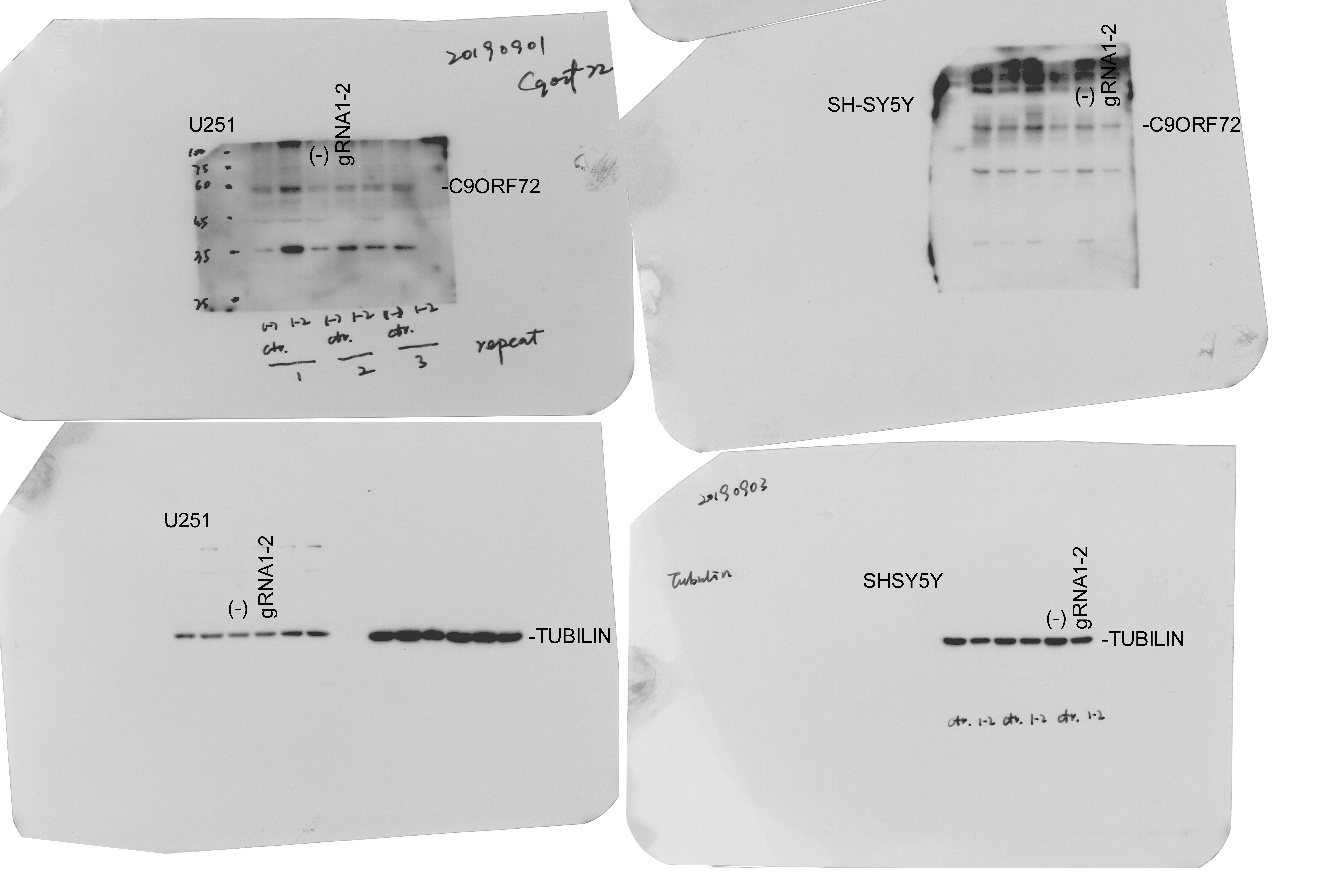
**

**Supplementary Fig. 26. Original uncropped Western blot images for Supplementary Fig. 8D.**

**
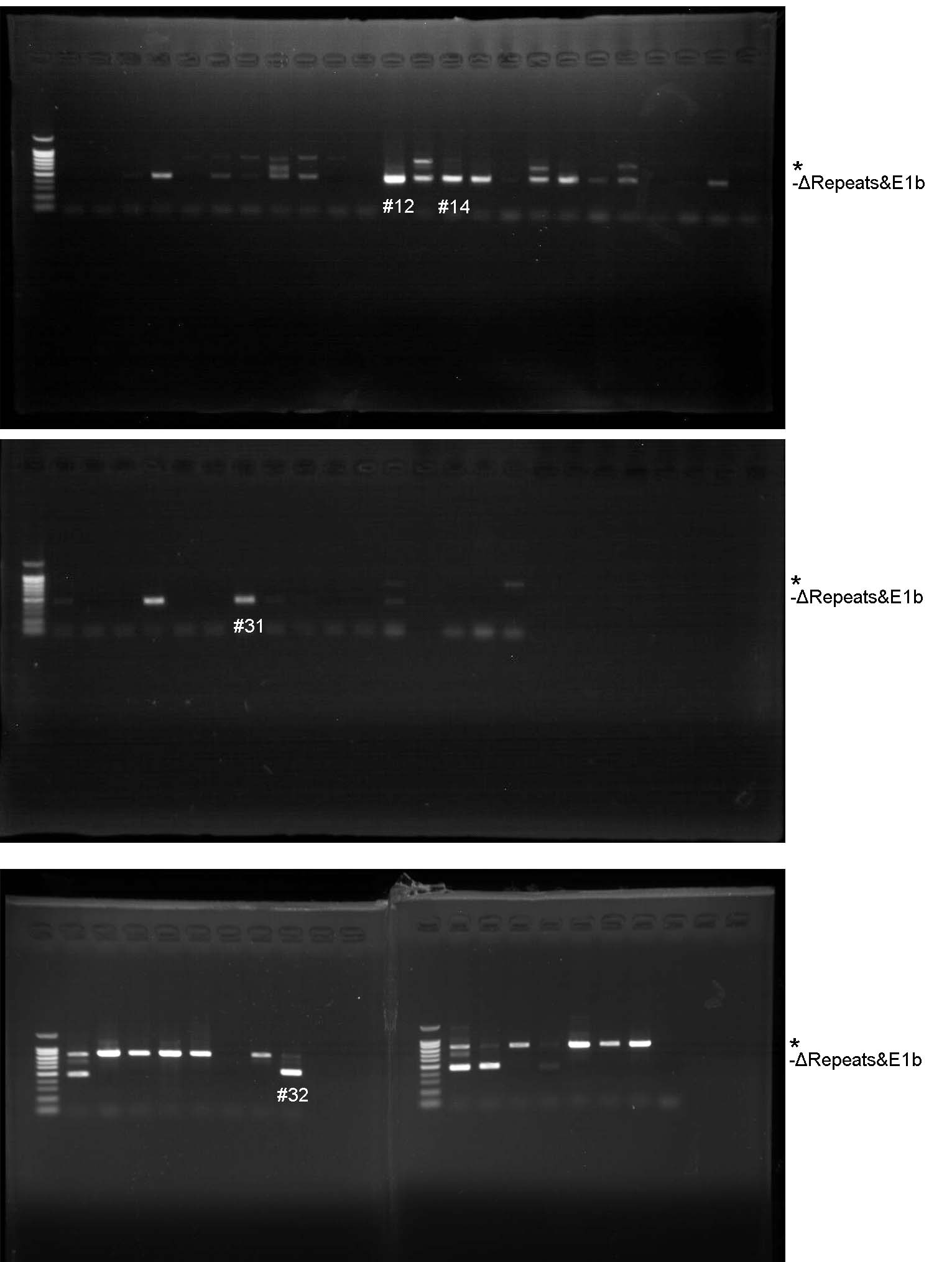
**

**Supplementary Fig. 27. Original uncropped DNA gel for Supplementary Fig. 9.**

| Supplementary Table 1. Off-target analysis of gRNAs for removal of *C9ORF72* repeat expansion. | | | | | | |
| --- | --- | --- | --- | --- | --- | --- |
| gRNA | Up-/down- stream of Ex1b | Exon (coding) | Exon +intron | Non-coding RNA | intergenic region | Total number of off-target sites |
| a | Downstream | 38 | 225 | 45 | 143 | 451 |
| b | Downstream | 14 | 52 | 13 | 33 | 112 |
| c | Downstream | 17 | 98 | 23 | 56 | 194 |
| d | Downstream | 107 | 700 | 103 | 370 | 1280 |
| e | Downstream | 29 | 192 | 46 | 94 | 361 |
| f | Downstream | 20 | 158 | 34 | 71 | 283 |
| g | Downstream | 58 | 370 | 63 | 166 | 657 |
| 1 | Upstream | 4 | 19 | 11 | 37 | 71 |
| 2 | Downstream | 1 | 12 | 0 | 6 | 19 |
| 3 | Upstream | 1 | 38 | 11 | 26 | 76 |
| Note: The online off-target predication programs employed for the purpose. The off-target sites were predicted by sgRNA Designer (http://portals.broadinstitute.org/gpp/public/analysis-tools/sgrna-design). The CFD (Cutting Frequency Determination) score we used between 0.2 to 1.0 (0.2≤CFD<1.0). | | | | | | |

| Supplementary Table 2. Numbers of gRNA1, gRNA2, gRNA3 and gRNAb off target sites predicted by Cas-OFFinder. | | | | | | | | |
| --- | --- | --- | --- | --- | --- | --- | --- | --- |
|  | Numbers of off-target sites | | | | | | | |
| gRNA | 0- | 1- | 2- | 3- | 4- | 5- | 6- | 7-mismatches |
| gRNA1 | 1 | 0 | 0 | 9 | 84 | 886 | 6500 | 41248 |
| gRNA2 | 1 | 0 | 0 | 1 | 21 | 326 | 3296 | 24889 |
| gRNA3 | 1 | 0 | 1 | 9 | 135 | 2146 | 7870 | 43595 |
| gRNAb | 1 | 0 | 1 | 43 | 303 | 2004 | 12532 | 59337 |
| Note: The off-target numbers were predicted by Cas-OFFinder (http://www.rgenome.net/cas-offinder/). | | | | | | | | |
